# Limb-Bone Scaling Indicates Diverse Stance and Gait in Quadrupedal Ornithischian Dinosaurs

**DOI:** 10.1371/journal.pone.0036904

**Published:** 2012-05-22

**Authors:** Susannah C. R. Maidment, Deborah H. Linton, Paul Upchurch, Paul M. Barrett

**Affiliations:** 1 Department of Palaeontology, Natural History Museum, London, United Kingdom; 2 Department of Earth Sciences, University College London, London, United Kingdom; Raymond M. Alf Museum of Paleontology, United States of America

## Abstract

**Background:**

The most primitive ornithischian dinosaurs were small bipeds, but quadrupedality evolved three times independently in the clade. The transition to quadrupedality from bipedal ancestors is rare in the history of terrestrial vertebrate evolution, and extant analogues do not exist. Constraints imposed on quadrupedal ornithischians by their ancestral bipedal bauplan remain unexplored, and consequently, debate continues about their stance and gait. For example, it has been proposed that some ornithischians could run, while others consider that none were cursorial.

**Methodology/Principal Findings:**

Drawing on biomechanical concepts of limb bone scaling and locomotor theory developed for extant taxa, we use the largest dataset of ornithischian postcranial measurements so far compiled to examine stance and gait in quadrupedal ornithischians. Differences in femoral midshaft eccentricity in hadrosaurs and ceratopsids may indicate that hadrosaurs placed their feet on the midline during locomotion, while ceratopsids placed their feet more laterally, under the hips. More robust humeri in the largest ceratopsids relative to smaller taxa may be due to positive allometry in skull size with body mass in ceratopsids, while slender humeri in the largest stegosaurs may be the result of differences in dermal armor distribution within the clade. Hadrosaurs are found to display the most cursorial morphologies of the quadrupedal ornithischian cades, indicating higher locomotor performance than in ceratopsids and thyreophorans.

**Conclusions/Significance:**

Limb bone scaling indicates that a previously unrealised diversity of stances and gaits were employed by quadrupedal ornithischians despite apparent convergence in limb morphology. Grouping quadrupedal ornithischians together as a single functional group hides this disparity. Differences in limb proportions and scaling are likely due to the possession of display structures such as horns, frills and dermal armor that may have affected the center of mass of the animal, and differences in locomotor behaviour such as migration, predator escape or home range size.

## Introduction

**Figure 1:**
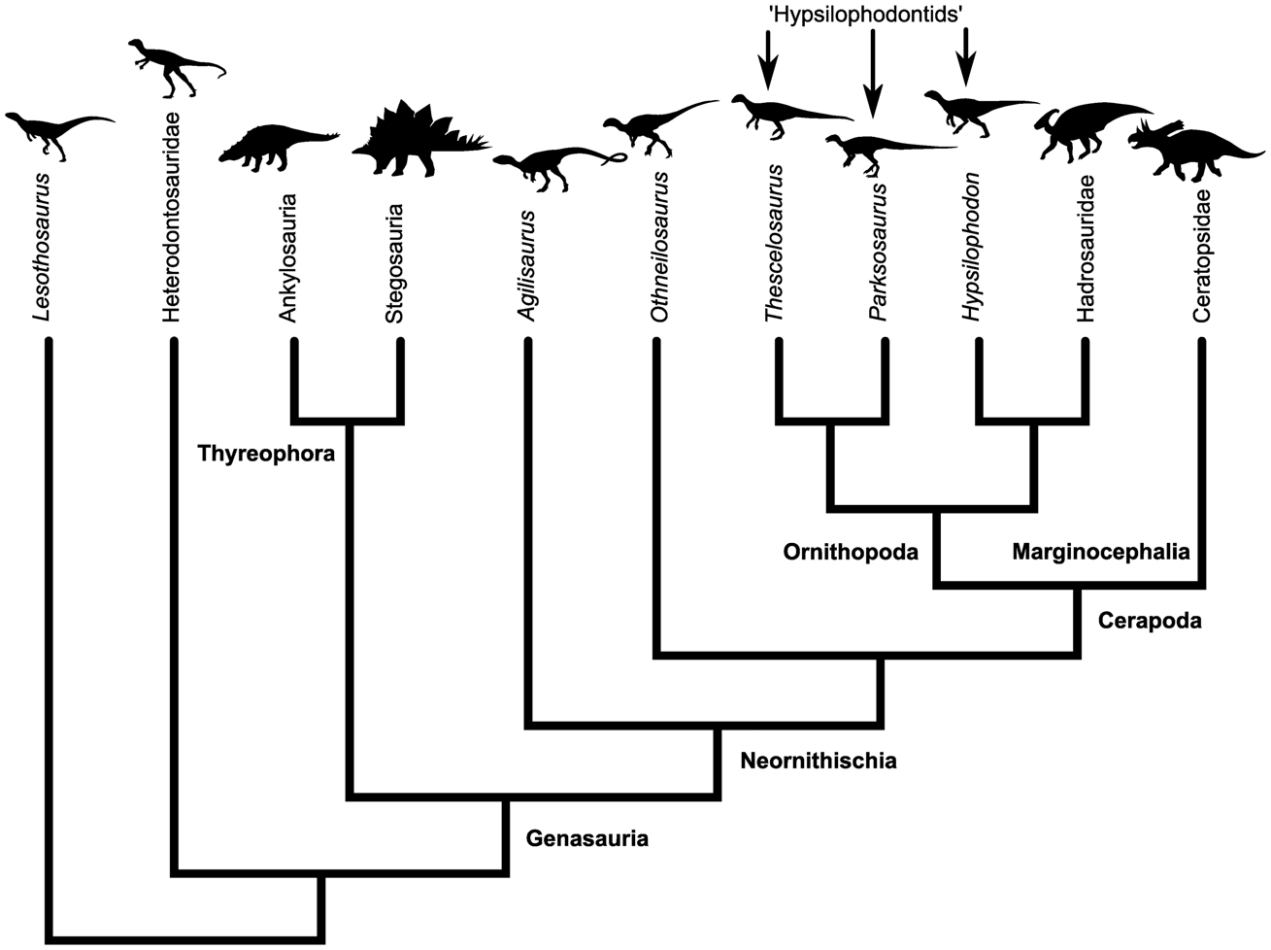
Simplified phylogeny of ornithischian dinosaur relationships, focusing on the taxa discussed in the text.

Ornithischia is a monophyletic clade of mainly herbivorous dinosaurs that dominated terrestrial ecosystems for much of the Mesozoic. Arising in the Late Triassic [1], primitive ornithischians were small (around 1.5 m long; e.g., [2], [3]) and bipedal, but during their 170 million year evolutionary history the clade diversified into a wide range of body shapes and sizes and quadrupedality evolved within at least three lineages independently: once in the armored stegosaurs and ankylosaurs, once in the frilled ceratopsids, and once in the duck-billed hadrosaurs (Fig. 1). Despite over50 years of debate regarding the stance and gait of ornithischian dinosaurs, little consensus has been reached. For example, some authors (e.g., [4], [5], [6]) have suggested that ceratopsids held their forelimbs in a crouched posture, with elbows orientated at an angle to the sagittal plane, while others (e.g., [7], [8]) proposed a more upright posture. Iguanodontids have been variously portrayed as bipeds (e.g., [9], [10], [11]), quadrupeds (e.g., [12], [13]) or somewhere in between (facultative quadrupeds, e.g., [14], [15], [16]). Bakker [7] suggested that some ornithischians would have been able to run, while Carrano [17] has stated that no ornithischian quadruped displayed the morphological indicators of cursoriality.

### Limb bone scaling

The body mass of an animal increases with the cube of limb element length, so the limb bones of large animals have to support a relatively much greater mass than those of small animals (e.g., [18]). In order to maintain bone safety factors, the limb bones of large animals are therefore expected to be much more robust (i.e. have a much greater circumference or diameter relative to their length) than those of small animals [19], [20]. Although initial studies of mammalian limb bone scaling with body mass suggested isometry across the entire size range displayed by extant taxa [21], more recent studies have shown differences between small and large taxa, and within specific mammalian clades [20], [22]. Small taxa are found to scale close to isometry, while larger taxa scale with positive allometry, having more robust limb bones than would be expected given their mass [20], [23].

Scaling rules developed using extant taxa (e.g., [20], [21], [23]) are based on the relationship between body mass and some linear dimension of a limb bone, such as length, diameter or circumference [18]. However, body mass is unknown for extinct taxa, and there is currently no single universally accepted method with which to estimate body mass for dinosaurs. Indeed, estimates of dinosaur body mass, even using the same methodology, often produce wildly different results. Mass estimates for the thyreophoran dinosaur *Stegosaurus* range from 1780 kg [24] to 3100 kg [25], both using scale models, while Henderson [26] produced a mass estimate of 2530 kg for *Stegosaurus* using a 3D computational model. Furthermore, mass estimates have only been published for a relatively limited number of ornithischians [24]–[27].

In extinct animals, the relationships between the linear dimensions of limb bones can be investigated to examine the relative differences in proportion between different taxa. Although this cannot be related to body mass, and theoretical expectations of geometric similarity or elastic similarity cannot be tested [18], bones respond to the forces acting upon them, so differences in stance, gait and locomotor style between taxa might be elucidated by differences in relative robustness.

Carrano [15], [17] used a large dataset of mammalian and dinosaurian taxa to investigate limb-bone scaling and its relationship to locomotion, and used several metrics to estimate mass in dinosaurs. He concluded that (a) dinosaurs and mammals have very similar limb scaling relationships with mass, pointing towards underlying mechanical or biological mechanisms influencing bone shape, (b) in general both mammalian and dinosaurian limb bones become slightly more robust than would be expected given isometric scaling with mass, (c) that in the hind limb, bipeds and quadrupeds have similar scaling patterns, and (d) the humeri of quadrupeds scale with isometry, while those of bipeds become more robust with mass. Carrano [15], [17] used a dataset that encompassed taxa representing all clades of Dinosauria, allowing general patterns to be established. Here, we use methods similar to those of Carrano [15] to examine ornithischian limb bone scaling patterns in detail, and in isolation from patterns that might be influenced by data from saurischians. This approach allows testing of hypotheses of stance and locomotor ability and utilizes a new, large dataset of limb bone measurements encompassing representatives of all ornithischian clades. Our study differs from that of Carrano [15] in that we group our taxa into phylogenetic rather than functional groups in order to examine similarities and differences between ornithischian bipeds and quadrupeds.

### Locomotor performance

The speed at which dinosaurs could have moved has generated a large amount of interest and research effort (e.g., [28]–[32]). Various techniques, from the examination of preserved tracks [28]–[30] to the application of evolutionary robotics [32], have been used to investigate the problem. However, maximum locomotor performance is difficult to measure even in extant taxa. Published maximum speeds for mammals have been attained using various methods and are of variable quality ([33], [34] and references therein), and although attempts have been made to identify osteological correlates for running speed, the results are far from conclusive (e.g., [33], [34]). Features thought to correlate with running speed have also been found to correlate with home range size, perhaps related to a reduction in transport costs during slow locomotion, for example [35].

Locomotor theory, however, has produced a series of predictions regarding differences in limb bone morphology that might exist between animals that move fast and those that move more slowly. The stance-phase limb can be considered as an inverted pendulum oscillating about the foot: a larger muscle mass located further from the center of rotation will decrease the period of oscillation, while lever mechanics show that muscles inserting closer to a joint will work at higher velocities, meaning that muscle insertions are more proximally located in taxa that move more quickly [17], [36]. Therefore taxa that possess a short, robust propodium, a long, slender epipodium, a fused, compact metapodium and muscle attachments located proximal to the body are considered to possess ‘cursorial’ morphologies [17], [37]. Cursorial morphology has not always been found to closely correlate with maximum running speed in extant mammals, and it may correspond with other features of locomotor performance, such as stamina or locomotor efficiency at slow speeds [33], [35]. Furthermore, the degree to which cursorial morphologies are expressed depends on taxon size, with small taxa tending to appear more ‘cursorial’ than large taxa [37].

Here, we compare the forelimbs of large quadrupedal or predominantly quadrupedal ornithischians to investigate which clade(s) developed the most cursorial morphology. We use forelimb morphology because Christiansen [34] examined a range of cursorial morphologies and found that the radius/humerus ratio correlated best with running speed in extant mammals. Clearly, the forelimbs of quadrupedal ornithischians are not geometrically similar to those of extant mammals. Quadrupedal ornithischians had habitually bent elbows and probably could not have protracted their humerus beyond vertical (Maidment and Barrett, unpublished data). The scapulae of mammals are mobile, contributing to effective forelimb length [38], while those of quadrupedal ornithischians were rigid and probably did not move significantly during the step cycle. The forelimbs are significantly shorter than the hind limbs in all quadrupedal ornithischians because they evolved from bipedal ancestors, which is not the case in mammals. These factors all indicate that ornithischians probably had shorter strides (being limited by forelimb length) than those of mammalian quadrupeds, and could not have moved as quickly. Despite these differences, predictions generated by locomotor theory suggest that radius/humerus ratio and the morphology of forelimb elements should be informative about relative locomotor performance in any terrestrial animal [36]. A relatively elongate and slender epipodium would be predicted in a terrestrial animal with relatively high locomotor performance, while a short and robust epipodium would be predicted in animals with lower relative locomotor performance [36], [37].

The metatarsal/femur ratio has also commonly been used to investigate correlations between cursorial morphology and locomotor performance (e.g., [33], [35], [39]) but the number of ornithischians for which we have data on both the femur and metatarsal (21 specimens) is too low to generate statistically meaningful results. Therefore, we focus on scaling of forelimb elements. Forelimb scaling will not allow us to directly investigate speed in large quadrupedal ornithischians, but it will allow us to compare relative locomotor abilities between the different clades.

### Aims

Using the concepts of limb bone scaling and locomotor theory outlined above, we aim to address the following hypotheses and questions:

1) All ornithischians, regardless of whether they were quadrupedal or bipedal, utilized the hind limbs primarily for locomotion, and in both bipeds and quadrupeds, centers of mass were located close to the hips [26]. There should therefore be no difference in femoral scaling between ornithischian bipeds and quadrupeds [15].

2) Ornithischian quadrupeds used the forelimbs primarily for weight support and locomotion, while in bipeds the forelimbs were adapted for other functions, such as grasping. Therefore, there should be differences in humeral scaling between ornithischian bipeds and quadrupeds [15].

3) Which clade of quadrupedal ornithischians displays the most cursorial morphology in the forelimb? What are the implications for locomotor performance in quadrupedal ornithischians?

## Materials and Methods

**Figure 2:**
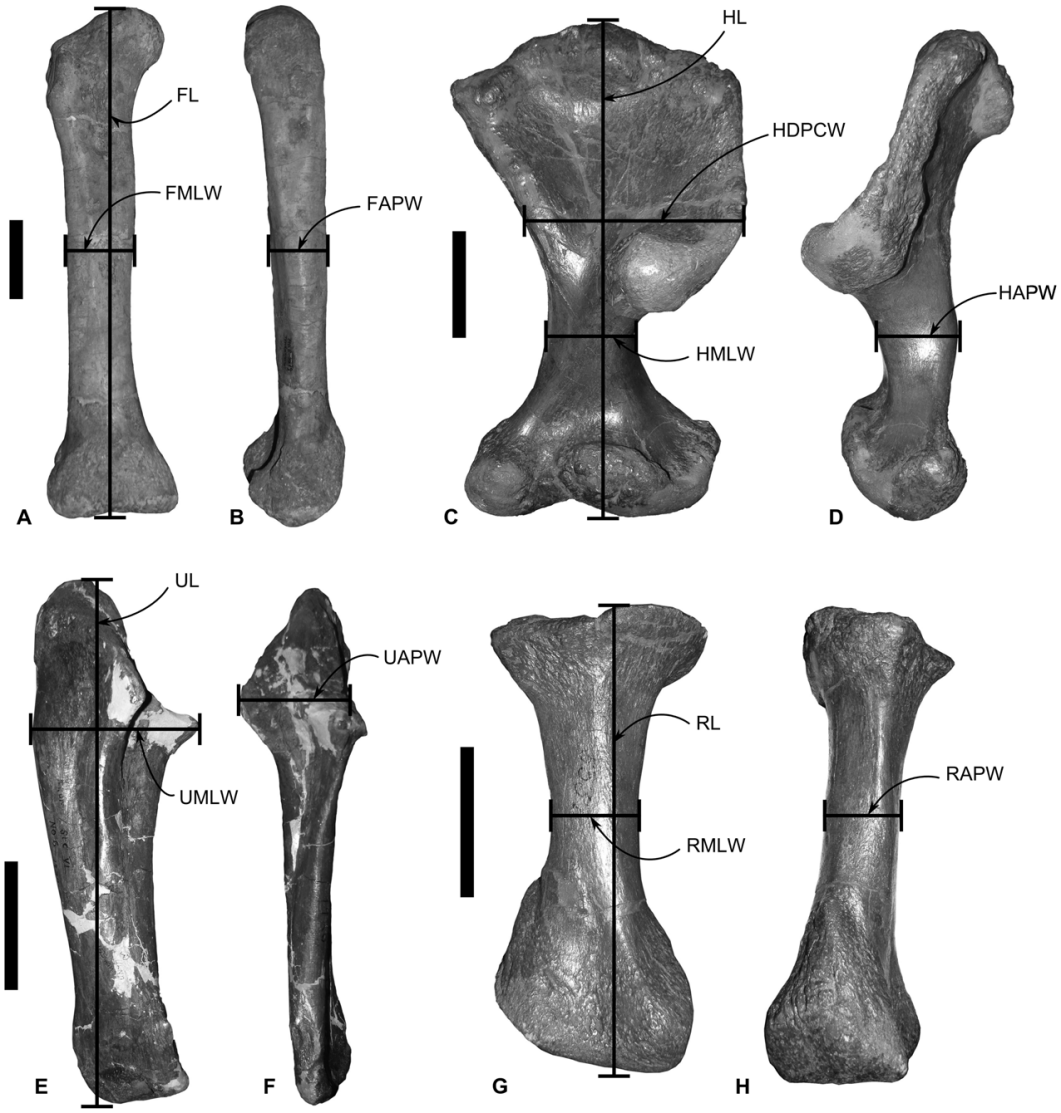
Measurements taken. A–B, right femur of the stegosaur *Kentrosaurus aethiopicus* (MB R.3759) in A, anterior and B, lateral view. C–D, left humerus of the ankylosaur *Euoplocephalus tutus* (AMNH [American Museum of Natural History, New York, USA] 5337) in C, anterior and D, lateral view. E–F, right ulna of the ceratopsid *Centrosaurus apertus* (ROM 1426) in E, anterior and F, medial view. G–H, right radius of the ankylosaur *Euoplocephalus tutus* (AMNH 5337) in G, anterior and H, lateral view. Abbreviations: **FAPW**, femur anteroposterior width; **FL**, femur length; **FMLW**, femur mediolateral width; **HAPW**, humerus anteroposterior width; **HDPCW**, humerus width across the deltopectoral crest; **HL**, humerus length; **HMLW**, humerus mediolateral width; **RAPW**, radius anteroposterior width; **RL**, radius length; **RMLW**, radius mediolateral width; **UAPW**, ulna anteroposterior width; **UL**, ulna length; **UMLW**, ulna mediolateral width. Scale bars equal to 10 cm.

Specimens used in this study and raw measurement data can be found in the Supporting Information S1 (SI). Measurements (Fig. 2) were taken on 250 ornithischian dinosaur specimens, representing 43 genera. Measurements under 15 cm were taken with callipers, while those over 15 cm were taken using a tape measure. Where data from left and right elements from the same specimen were taken, the measurements were averaged. When particular groups had very small sample sizes, direct measurement data were supplemented by measurements taken from photographs. Data from specimens that had clearly suffered post-mortem deformation were excluded. Because this study is focused on interspecific and inter-clade allometric variation, it is best practise to calculate average measurements for each species rather than include each data point from every individual specimen [40]. This prevents the introduction of intraspecific variation into the analysis, which may swamp interspecific patterns. However, this is rarely practicable for vertebrate paleontological studies where sample sizes are usually very low. The inclusion of average species values in this study would result in too few data points for any statistically significant patterns to emerge. Following previous studies (e.g., [15], [17]) data from each individual specimen measured was used in regressions, but to limit the effect of ontogenetic variation, we excluded any specimen smaller than 50% the size of the largest specimen of the same species. We acknowledge that this methodology may result in intraspecific patterns swamping interspecific variation, but this is unavoidable until larger sample sizes are available. Linear data were log-transformed before analysis.

### Groups used in analyses

The aim of this study was to examine limb-bone scaling differences between bipedal and quadrupedal ornithischians, and to investigate scaling differences between different clades of quadrupedal ornithischians. It was therefore necessary to (a) identify taxa in which stance was uncontroversial and (b) group taxa into monophyletic groups. Ankylosauria, Stegosauria, and Ceratopsidae are monophyletic clades of ornithischians that represent ‘crown group’ radiations of Thyreophora and Marginocephalia respectively (Fig. 1). Members of Ankylosauria, Stegosauria and Ceratopsidae are all uncontroversially quadrupedal. Hadrosauridae is the ‘crown group’ radiation of Ornithopoda (Fig. 1). Although stance in hadrosaurs has been controversial (see Introduction), there is now a growing consensus, based on postcranial anatomy ([13]; ‘Predominantly quadrupedal hadrosaurs’ below), soft tissue preservation [41] and trackways [12], that hadrosaurs were predominantly quadrupedal. Postcranial anatomy within the group is relatively conservative (SCRM pers. obs. 2009–2011) showing that all members of the clade were locomoting in a similar manner. Taxa included in these groups follow recent phylogenetic analyses [42]–[46].

In order to compare limb-bone scaling between these quadrupeds and bipedal taxa, a number of uncontroversially bipedal ornithischians were grouped together into a polyphyletic group which included heterodontosaurids (e.g. *Heterodontosaurus*, *Abrictosaurus*), the basal ornithischian *Lesothosaurus,* non-cerapodan neornithischians (e.g. *Agilisaurus*; *Othneilosaurus* ) and non-iguanodontian ornithopods (*sensu* [47]; often described as ‘hypsilophodontids’; e.g. *Thescelosaurus, Parksosaurus*, *Hypsilophodon*; Fig. 1). Bipedality is the basal condition for ornithischians and to our knowledge none of these taxa have ever been considered anything but bipedal in the literature (e.g., [48], [49]).

Stance in other ornithischians, including non-eurypodan thyreophorans, some non-hadrosaurid ornithopods and non-ceratopsid marginocephalians, is more controversial (e.g., [14], [50]–[53]). Furthermore, some members of these ‘stem’ lineages are more closely related to the ‘crown’ than others, and features relating to quadrupedality were acquired in a step-wise fashion as the ‘crown group’ was approached (Maidment and Barrett, unpublished data). The inclusion of ‘stem’ taxa may therefore obscure differences in limb-bone scaling between bipedal and quadrupedal taxa. However, for completeness, taxa were also grouped into the larger phylogenetic groups Thyreophora, Ornithopoda and Marginocephalia. Taxa in these groups followed the phylogeny of Butler et al. [1]. This increased sample sizes and allowed the assessment of phylogenetic trends.

### Reduced major axis regression

We analysed scaling relationships of the femora, humeri, radii, and ulnae in order to examine the questions and hypotheses outlined in the Introduction. Reduced major axis (RMA) regression was used to explore allometric differences in limb bone scaling between the phylogenetic groups. RMA regression was chosen as it assumes both variables are independent and subject to error [54]. RMA regressions were carried out in the freeware paleontological statistics package PAST (v. 2.09; http://folk.uio.no/ohammer/past; [55]), which outputs the allometric coefficient, along with the probability that the slope of the line differs significantly from isometry. Analyses of covariance (ANCOVA) were then used to examine the homogeneity of slopes and differences between population means. Pairwise ANCOVAs were carried out to compare slopes and population means between all pairs of phylogenetic groups. ANCOVA assumes normality of data and equality of variances. As is expected in a paleontological dataset, a Shapiro-Wilk test confirmed that the majority of the data were not normally distributed. However, variances are similar across all data series, and so are sample sizes. Simulations suggest ANCOVA is robust to violations of normality as long as variances are not too dissimilar [56], [57], so it is an appropriate statistical method to use here.

The use of ANCOVAs to compare slopes and population means between all possible pairs of taxa is not statistically sound because the chances of recovering a statistically significant result at the p = 0.05 level is greatly increased (a Type I error; [54]). A post-hoc Bonferroni correction was therefore applied to the significance level of all pairwise comparisons before rejection of the null hypothesis.

### Independent contrasts

An assumption of any regression analysis is that the data points are independent of one another; however, in biological systems this assumption is violated because the data points are hierarchically linked by phylogeny and therefore are not truly independent (e.g., [58], [59]). The method of Independent Contrasts [39], [58], [59] was therefore used to remove the effects of phylogeny on the dataset, and was implemented in the freeware comparative analysis software CAIC (v. 2.6.9: http://www.bio.ic.ac.uk/evolve/software/caic/; [60]).

Recent phylogenetic analyses of Ornithischia and its constituent clades were used [1], [42]–[47], [61]–[63]. The phylogenetic position of all data points in the analysis must be known, so specimens indeterminate to species level (see SI) and those that were not included in the above phylogenetic analyses (*Dyoplosaurus acutosquameus* ROM [Royal Ontario Museum, Toronto, Canada] 784; *Fulgotherium australe* NHMUK [Natural History Museum, London, UK] R12209; *Hoplitosaurus marshi* USNM [National Museum of Natural History, Smithsonian Institution, Washington D.C., U.S.A.] 4752; *Jeholosaurus shanguyuanensis* IVPP [Institute of Vertebrate Paleontology and Paleoanthropology, Beijing, People's Republic of China] V15939; *Nipponsaurus sachalinensis* NMST [National Science Museum of Tokyo, Japan] *Nipponsaurus*) were excluded.

Branch lengths used were divergence times [33], [40] based on the first appearance of the taxon in the fossil record. Stratigraphic data for all species was derived from Weishampel et al. [64]. The resolution with which various specimens, species and formations are dated varies widely across Ornithischia, so the first appearance datum of each species was assigned an age based on the maximum age of the formation in which the species is found (see SI), regardless of whether or not the specimen could be dated more accurately. The timescale used was that of Ogg et al. [65]. The minimum branch length possible in CAIC is 2, so 2 was added to all branch lengths before analysis.

Contrasts are calculated by subtracting trait values in sister taxa or from the value at the node, and the resulting raw contrasts are standardized by division by the square root of the sum of the branch length for each contrast [40], [58], [59]. An assumption of the method is that there should be no correlation between the standardized contrasts and their standard deviations, indicating that the contrasts have been brought to common variance and there is no relationship with branch lengths [39]. Several of the initial analyses violated this assumption, so the branch lengths were logarithmically transformed [39], and 2 was added to the transformed data once again.

Standardized contrasts for traits of interest were then regressed in PAST [55] using RMA regressions forced through the origin [59], and the allometric coefficient and probability that the slope of the line differed from isometry were recorded. Because Independent Contrasts proceeds by subtracting the values of sister taxa from each other to produce a nodal value, the sample size is significantly reduced [58]. Where a polytomy exists in a phylogeny sample sizes are reduced further because there is only one node for a number of specimens. Although it is favourable to perform a technique that takes into account phylogeny when investigating function through scaling, a large sample size is needed to generate a meaningful or significant result. The dataset used in this study represents, to our knowledge, the largest compilation of ornithischian postcranial measurements available; however, sample sizes for many groups are still extremely small (e.g., Ankylosauria, femora: n = 7). Thus, the results of the Independent Contrasts analyses are frequently not statistically significant, and in many cases no correlation could be found between the X and Y variables at the p = 0.05 level (Tables 1, 2, 3, 4). We consider this to be due to the very small sample size rather than a real biological signal, and further work to increase the sample sizes in this study would clearly be favorable. Therefore we report the results of the Independent Contrasts analyses, but interpret them with caution. Basal bipedal ornithischians were not examined using Independent Contrasts because they were grouped on functional rather than phylogenetic grounds and represent a polyphyletic group. Any phylogeny produced for this group would therefore be incomplete.

**Table 1:** RMA regressions of raw data and standardized independent contrasts for the femur.

	RMA Regression: Raw data						RMA Regression: Independent Contrasts				
FLFAPW	n	R-squared	p(uncorr)	a	b	p(a = 1)	n	R-squared	p(uncorr)	a	p(a = 1)
Ankylosauria	7	0.577	0.047	1.455	−1.998	0.331	2	–	–	–	–
Stegosauria	19	0.303	0.015	0.817	−0.539	0.284	5	0.008	**0.889**	0.679	0.293
Hadrosauridae	21	0.794	0	1.618	−2.708	**0.002**	11	0.348	**0.056**	1.35	0.235
Ceratopsidae	14	0.516	0.004	1.537	−2.451	0.107	6	0.001	**0.965**	1.572	0.343
Bipeds	16	0.97	0	1.091	−1.111	0.09	–	–	–	–	–
Thyreophora	27	0.169	0.033	0.821	−0.482	0.242	9	0.371	**0.082**	0.667	0.046
Ornithopoda	57	0.921	0	1.112	−1.187	**0.01**	29	0.623	0	1.208	0.059
Marginocephalia	17	0.646	0	1.192	−1.45	0.312	9	0.105	0.395	1.121	0.643
Ornithischia	107	0.841	0	1.049	−1.053	0.228	55	0.54	0	1.113	0.139
**FLFMLW**											
Ankylosauria	7	0.963	0	1.205	−1.291	0.106	2	–	–	–	–
Stegosauria	19	0.834	0	0.976	−0.786	0.804	5	0.946	0.005	0.977	0.853
Hadrosauridae	21	0.757	0	1.115	−1.246	0.373	11	0.396	0.038	1.381	0.212
Ceratopsidae	14	0.838	0	1.667	−2.757	**0.005**	6	0.837	0.011	1.989	–
Bipeds	16	0.939	0	1.301	−1.624	**0.004**	–	–	–	–	
Thyreophora	27	0.842	0	0.912	−0.581	0.238	9	0.885	0	1.15	0.159
Ornithopoda	57	0.95	0	1.117	−1.207	0.001	29	0.734	0	1.207	**0.023**
Marginocephalia	17	0.882	0	1.365	−1.878	**0.008**	9	0.679	0.006	1.451	0.067
Ornithischia	107	0.945	0	1.151	−1.285	**0**	55	0.752	0	1.242	**0**

Abbreviations: **FLFAPW**, femoral length against anteroposterior width; **FLFMLW**; femoral length against mediolateral width; **a**, allometric coefficient; **b**, y-intercept; **n**, sample size; **p(a = 1)**, probability that the allometric coefficient is equal to isometry, cells highlighted in bold are those that are statistically distinguishable from isometry at the p = 0.05 level; **p(uncorr)**, probability that X and Y are uncorrelated, cells highlighted in bold are those where X and Y are uncorrelated at the p = 0.05 level; **R-squared**, coefficient of determination.

**Table 2:** RMA regressions of raw data and standardized independent contrasts for the humerus.

	RMA regression: Raw data						RMA regression: Independent Contrasts				
HLHAPW	n	R-squared	p(uncorr)	a	b	p(a = 1)	n	R-squared	p(uncorr)	a	p(a = 1)
Ankylosauria	12	0.035	0.559	1.658	−2.644	0.23	6	0.201	**0.372**	1.114	0.814
Stegosauria	17	0.763	0	0.974	−0.765	0.835	6	0.6	**0.071**	0.774	0.121
Hadrosauridae	35	0.795	0	1.118	−1.324	0.191	15	0.786	0	1.120	0.232
Ceratopsidae	23	0.807	0	1.435	−2.171	**0.005**	7	0.329	**0.178**	1.059	0.798
Bipeds	11	0.968	0	1.081	−1.217	0.241	–	–	–	–	–
Thyreophora	29	0.545	0	1.137	−1.224	0.362	13	0.579	0.003	0.823	0.196
Ornithopoda	69	0.892	0	1.068	−1.181	0.118	31	0.792	0	1.062	0.297
Marginocephalia	28	0.88	0	1.211	−1.554	**0.016**	12	0.738	0	1.116	0.335
Ornithischia	129	0.867	0	1.173	−1.421	**0**	61	0.806	0	1.077	0.082
**HLHMLW**											
Ankylosauria	13	0.476	0.009	1.148	−1.17	0.567	7	0.91	0.001	1.097	0.619
Stegosauria	17	0.789	0	0.923	−0.522	0.495	6	0.755	0.025	0.688	**0.012**
Hadrosauridae	35	0.808	0	1.118	−1.169	0.178	15	0.523	0.002	1.073	0.627
Ceratopsidae	23	0.906	0	1.414	−1.962	0	7	0.786	0.008	1.278	0.084
Bipeds	11	0.985	0	1.19	−1.387	0.003	–	–	–	–	–
Thyreophora	30	0.684	0	1.06	−0.91	0.601	14	0.702	0	0.836	0.096
Ornithopoda	69	0.929	0	1.192	−1.371	0	31	0.683	0	1.118	0.145
Marginocephalia	28	0.953	0	1.322	−1.71	0	12	0.909	0	1.282	**0.003**
Ornithischia	130	0.922	0	1.272	−1.554	0	62	0.751	0	1.156	**0.004**
**HLHDPCW**											
Ankylosauria	13	0.161	**0.174**	1.586	−1.997	0.208	7	0.023	**0.747**	1.372	0.413
Stegosauria	16	0.598	0	1.068	−0.555	0.714	6	0.592	**0.128**	0.888	0.756
Hadrosauridae	35	0.878	0	1.133	−0.949	0.062	15	0.925	0	1.083	0.151
Ceratopsidae	23	0.918	0	1.361	−1.472	**0**	7	0.932	0	1.219	**0.03**
Bipeds	11	0.956	0	1.214	−1.211	**0.033**	–	–	–	–	–
Thyreophora	29	0.453	0	1.317	−1.254	0.103	13	0.206	**0.12**	1.071	0.695
Ornithopoda	69	0.918	0	1.227	−1.227	**0**	31	0.874	0	1.192	**0.001**
Marginocephalia	28	0.934	0	1.281	−1.296	**0**	12	0.817	0	1.172	0.153
Ornithischia	129	0.867	0	1.357	−1.498	**0**	61	0.829	0	1.219	**0**

Abbreviations: **HLHAPW**, humeral length against anteroposterior width; **HLHMLW**; humeral length against mediolateral width; **HLHDPCW**, humeral length against deltopectoral crest width; **a**, allometric coefficient; **b**, y-intercept; **n**, sample size; **p(a = 1)**, probability that the allometric coefficient is equal to isometry, cells highlighted in bold are those that are statistically distinguishable from isometry at the p = 0.05 level; **p(uncorr)**, probability that X and Y are uncorrelated, cells highlighted in bold are those where X and Y are uncorrelated at the p = 0.05 level; **R-squared**, coefficient of determination.

**Table 3:** RMA regressions of raw data and standardized independent contrasts for the radius.

	RMA regression: Raw data						RMA regression: Independent Contrasts				
RLRAPW	n	R-squared	p(uncorr)	a	b	p(a = 1)	n	R-squared	p(uncorr)	a	p(a = 1)
Ankylosauria	8	0.608	0.022	0.846	−0.387	0.501	5	0.003	**0.928**	0.642	0.173
Stegosauria	10	0.552	0.014	1.064	−0.983	0.806	2	–	–	–	–
Hadrosauridae	28	0.885	0	1.156	−1.541	**0.053**	18	0.839	0	1.078	0.376
Ceratopsidae	14	0.285	0.048	0.976	−0.736	0.921	7	0.814	0.005	1.504	0.062
Bipeds	5	0.931	0.008	1.32	−1.644	0.208	–	–	–	–	–
Thyreophora	18	0.653	0	0.848	−0.405	0.24	8	0.04	**0.634**	0.656	0.08
Ornithopoda	51	0.801	0	1.035	−1.132	0.603	32	0.872	0	1.081	0.137
Marginocephalia	17	0.601	0	1.344	−1.66	0.138	10	0.936	0	1.595	**0.002**
Ornithischia	88	0.671	0	1.14	−1.303	**0.051**	54	0.815	0	1.157	**0.004**
**RLRMLW**											
Ankylosauria	8	0.154	**0.336**	−0.74	3.711	0.001	5	0.003	**0.933**	−0.65	0.006
Stegosauria	10	0.107	**0.357**	1.456	−1.854	0.376	2	–	–	–	–
Hadrosauridae	28	0.764	0	1.223	−1.587	0.067	18	0.542	0	1.303	0.105
Ceratopsidae	14	0.04	**0.493**	1.422	−1.878	0.315	7	0.137	**0.413**	1.985	0.19
Bipeds	5	0.605	**0.122**	0.743	−0.344	0.411	–	–	–	–	–
Thyreophora	18	0.062	**0.321**	1.012	−0.688	0.962	8	0.028	**0.695**	−0.61	0
Ornithopoda	51	0.769	0	1.089	−1.152	0.242	32	0.594	0	1.171	0.118
Marginocephalia	17	0.263	0.035	1.305	−1.581	0.308	10	0.362	**0.066**	1.511	0.177
Ornithischia	88	0.6	0	1.16	−1.244	**0.051**	54	0.532	0	1.168	0.07

Abbreviations: **RLRAPW**, radial length against anteroposterior width; **RLRMLW**; radial length against mediolateral width; **a**, allometric coefficient; **b**, y-intercept; **n**, sample size; **p(a = 1)**, probability that the allometric coefficient is equal to isometry, cells highlighted in bold are those that are statistically distinguishable from isometry at the p = 0.05 level; **p(uncorr)**, probability that X and Y are uncorrelated, cells highlighted in bold are those where X and Y are uncorrelated at the p = 0.05 level; **R-squared**, coefficient of determination.

**Table 4:** RMA regressions of raw data and standardized independent contrasts for the ulna.

	RMA regression: Raw data						RMA regression: Independent Contrasts				
ULUAPW	n	R-squared	p(uncorr)	a	b	p(a = 1)	n	R-squared	p(uncorr)	a	p(a = 1)
Ankylosauria	6	0.548	**0.092**	4.399	−9.459	0.083	5	0.38	**0.268**	4.919	0.06
Stegosauria	7	0.185	**0.335**	1.534	−2.056	0.428	2	–	–	–	–
Hadrosauridae	31	0.647	0	1.118	−1.163	0.345	16	0.713	0	1.198	0.156
Ceratopsidae	16	0.549	0.001	1.904	−3.141	**0.019**	7	0.204	**0.309**	1.214	0.461
Bipeds	5	0.966	0.003	1.354	−1.519	0.092	–	–	–	–	–
Thyreophora	13	0.323	0.043	2.488	−4.562	**0.035**	8	0	**0.98**	−3.12	0.003
Ornithopoda	60	0.803	0	1.049	−0.908	0.43	31	0.772	0	1.202	**0.011**
Marginocephalia	21	0.731	0	1.358	−1.655	**0.039**	9	0.544	0.023	1.364	0.175
Ornithischia	95	0.743	0	1.167	−1.181	**0.008**	53	0.637	0	1.281	**0.001**
**ULUMLW**											
Ankylosauria	6	0.567	**0.084**	1.398	−1.419	0.436	5	0.132	**0.548**	1.761	0.28
Stegosauria	7	0.497	**0.077**	0.763	0.185	0.374	2	–	–	–	–
Hadrosauridae	31	0.804	0	1.021	−0.781	0.807	16	0.893	0	1.036	0.699
Ceratopsidae	16	0.937	0	1.286	−1.206	**0.005**	7	0.887	0.002	1.228	**0.049**
Bipeds	5	0.967	0.003	1.239	−1.121	0.162	–	–	–	–	–
Thyreophora	13	0.447	0.013	0.88	−0.102	0.554	8	0.024	**0.717**	1.118	0.718
Ornithopoda	60	0.837	0	0.95	−0.528	0.322	31	0.865	0	1.083	0.132
Marginocephalia	21	0.971	0	1.194	−0.956	**0.001**	9	0.923	0	1.156	0.149
Ornithischia	95	0.754	0	1.104	−0.848	**0.071**	53	0.853	0	1.121	**0.008**

Abbreviations: **ULUAPW**, ulnar length against anteroposterior width; **ULUMLW**; ulnar length against mediolateral width; **a**, allometric coefficient; **b**, y-intercept; **n**, sample size; **p(a = 1)**, probability that the allometric coefficient is equal to isometry, cells highlighted in bold are those that are statistically distinguishable from isometry at the p = 0.05 level; **p(uncorr)**, probability that X and Y are uncorrelated, cells highlighted in bold are those where X and Y are uncorrelated at the p = 0.05 level; **R-squared**, coefficient of determination.

### Cluster analyses

Ward's cluster analysis and K-means cluster analysis were used to examine similarities and differences in forelimb proportions between the phylogenetic groups in PAST [55]. The humerus to radius ratio was calculated for each specimen. Ward's cluster analysis uses the Euclidean distance, the linear distance between two points in multidimensional space, to cluster taxa into a dendrogram [54]. K-means cluster analysis requires the user to specify the number of clusters *a priori*. Four clusters were specified, in order to examine whether phylogenetic groups (basal ornithischians, Thyreophora, Marginocephalia, Ornithopoda) cluster separately. K-means cluster analysis iteratively reassigns data points to clusters in order to produce clusters with the lowest variance possible [54]. K-means cluster analysis is not guaranteed to produce the same result each time it is carried out [54], so it was run five times independently. Pairwise ANCOVAs of radius length plotted against humerus length were used to examine whether the adjusted means of each phylogenetic group could be distinguished from the others.

### Predominantly quadrupedal hadrosaurs

Throughout this study, we assume that hadrosaurs were predominantly quadrupedal. Numerous lines of osteological evidence suggest that hadrosaurs used their forelimbs for weight-bearing, and could not have utilized them for grasping. The proximal ulna of hadrosaurs bears an anterolateral process (e.g., *Corythosaurus* ROM 1947, *Edmontosaurus* ROM 801; *Brachylophosaurus* CMN [Canadian Museum of Nature, Ottawa, Canada] 8893; [66]–[68]), a feature that is also present in all other quadrupedal ornithischians (ceratopsids, stegosaurs and ankylosaurs) and quadrupedal sauropodomorphs [69]. This means that the radius articulates with the ulna anteromedially at the proximal end. Distally, the radius is also located medial to the ulna, indicated by a concave, cup-like facet medially on the ulna (*Brachylophosaurus* CMN 8893; *Lambeosaurus* ROM 1218; [68]), and a facet laterally on the radius (*Brachlophosaurus* CMN 8893). This condition contrasts with that in basal dinosaurs [69] and bipedal ornithischians, where the radius was located anterior to the ulna (e.g., *Dysalotosaurus* MB [Museum für Naturkunde, Berlin, Germany] R.1408; *Hypsilophodon* NHMUK R196), causing the manus to be supinated when articulated; a feature presumably required for manual manipulation of food and grasping. Medial movement of the radius in hadrosaurs and other quadrupedal ornithischians would have resulted in pronation of the manus, which is required for quadrupedal locomotion [69]. The morphology of the distal articular facets of the ulna and radius in hadrosaurs precludes rotation of the distal end of the radius around the ulna, so that supination of the manus would have been impossible [69].

It has been suggested that the reduction of the hadrosaurian carpus to a pair of small bones indicates that the wrist was relatively weak and not capable of supporting weight [9]. However, the carpus is much reduced in all groups of uncontroversial quadrupedal ornithischians: adult stegosaurs have two carpals [70], ossified carpal elements are unknown in ankylosaurs [71] and four small, flat carpals are known in ceratopsids [72]. The number of carpal elements is also progressively reduced during the evolutionary transition from bipedal basal sauropodomorphs to undoubtedly quadrupedal sauropods [73]–[75], and derived forms such as titanosaurs frequently lack ossified carpals [76], [77]. Reduction in carpal ossification cannot therefore be used to preclude a quadrupedal stance in hadrosaurs.

Metacarpals II–IV of the hadrosaurian manus are elongate and closely appressed (*Edmontosaurus* ROM 801; *Corythosaurus* ROM 845; [13], [43], [78]). Proximal phalanges are columnar and cylindrical, while the distal ends of the metacarpals and all phalanges lack roller joints (*Edmontosaurus* ROM 801; [13], [66]). The unguals of digits II and III are hoof-like [66], [67], a feature observed in all other obligate quadrupedal ornithischians (SCRM pers. obs. 2009–2011). The columnar and tightly appressed nature of the metacarpals and proximal phalanges suggests that they functioned as a single unit. The lack of roller joints between phalanges suggests that flexing of the digits would not have been possible [13]. Finally, the hoof-like unguals are clearly adapted for weight-bearing. These features were also used to indicate a weight-bearing function in the facultatively quadrupedal ornithopod *Iguanodon bernissartensis* [14].

The pronation and weight-bearing adaptations of the manus suggests that adult hadrosaurs were predominantly quadrupedal [13]. This osteological evidence is supported by the numerous reports of quadrupedal hadrosaur trackways (e.g., [12]) and abrasion calluses on the manus observed in exceptionally preserved specimens [41].

## Results

Allometric coefficients and p values for both RMA regressions and Independent Contrasts are shown in Tables 1, 2, 3, 4. The results of ANCOVA pairwise comparisons are shown in Tables 5, 6, 7, 8, 9.

**Table 5:** Pairwise ANCOVAs of the femur.

	FLFAPW		FLFMLW	
Taxon pair	p(same)	p(HoS)	p(same)	p(HoS)
**Ank vs Cer**	0.083	0.998	**0.001**	0.228
**Ank vs Had**	0.005	0.367	**0.000**	0.349
**Ank vs Steg**	0.004	0.072	**0.001**	0.060
**Ank vs Bi**	0.177	0.907	0.029	0.686
**Ank vs Mar**	0.063	0.806	**0.001**	0.628
**Ank vs Orn**	0.029	0.893	**0.000**	0.650
**Cer vs Had**	0.907	0.307	0.065	0.017
**Cer vs Steg**	0.007	0.065	0.675	0.005
**Cer vs Bi**	0.392	0.908	0.114	0.186
**Cer vs Thy**	0.172	0.019	0.324	**0.000**
**Cer vs Orn**	0.163	0.871	0.541	0.013
**Had vs Steg**	**0.000**	**0.000**	0.015	0.657
**Had vs Bi**	0.041	0.036	0.312	0.065
**Had vs Mar**	0.864	0.088	0.049	0.069
**Had vs Thy**	0.024	**0.000**	**0.002**	0.340
**Steg vs Bi**	0.065	**0.000**	0.709	0.018
**Steg vs Mar**	0.005	0.062	0.904	0.588
**Steg vs Orn**	0.000	0.006	0.385	0.243
**Bi vs Mar**	0.557	0.685	0.287	0.775
**Bi vs Thy**	0.212	**0.000**	0.043	**0.000**
**Mar vs Thy**	0.296	0.007	0.201	**0.001**
**Orn vs Mar**	0.189	0.655	0.569	0.062
**Orn vs Thy**	0.003	**0.000**	0.006	0.011

Abbreviations: **FLFAPW**, femur length against anteroposterior width; **FLFMLW**, femur length against mediolateral width; **Ank**, Ankylosauria; **Bi**, basal bipedal ornithischians; **Cer**, Ceratopsidae; **Had**, Hadrosauridae; **Mar**, Marginocephalia; **Orn**, Ornithopoda; **Steg**, Stegosauria; **Thy**, Thyreophora; **p(same)**, the probability that the adjusted means of the two groups differ, cells highlighted in bold are those comparisons that can be statistically distinguished at the p = 0.002 level; **p(HoS)**, the probability that the slopes of the two groups are the same, cells highlighted in bold are those comparisons that can be statistically distinguished at the p = 0.002 level. P = 0.002 is the significance level applied using the Bonferroni correction for multiple pairwise comparisons.

**Table 6:** Pairwise ANCOVAs of the humerus.

	HLHAPW		HLHMLW		HLHDPCW	
Taxon pair	p(same)	p(HoS)	p(same)	p(HoS)	p(same)	p(HoS)
**Ank vs Cer**	0.003	0.152	0.007	0.017	0.003	0.114
**Ank vs Had**	**0.000**	0.274	0.009	0.173	**0.000**	0.246
**Ank vs Steg**	0.041	0.243	0.018	0.084	0.039	0.508
**Ank vs Bi**	0.011	**0.011**	0.003	0.006	**0.002**	0.228
**Ank vs Mar**	0.004	0.203	0.004	0.017	0.003	0.162
**Ank vs Orn**	0.000	0.218	**0.001**	0.060	**0.000**	0.189
**Cer vs Had**	0.738	0.079	0.788	0.016	0.020	0.053
**Cer vs Steg**	**0.000**	0.068	**0.000**	0.004	**0.000**	0.019
**Cer vs Bi**	0.484	0.171	0.947	0.149	0.026	0.386
**Cer vs Thy**	0.000	0.061	**0.000**	0.002	**0.000**	0.048
**Cer vs Orn**	0.962	**0.029**	0.588	0.066	**0.000**	0.320
**Had vs Steg**	**0.000**	0.482	0.000	0.370	**0.000**	0.226
**Had vs Bi**	0.843	0.629	0.089	0.157	0.020	0.265
**Had vs Mar**	0.556	0.272	0.850	0.008	0.060	0.074
**Had vs Thy**	**0.000**	0.487	**0.000**	0.290	**0.000**	0.323
**Steg vs Bi**	**0.000**	0.145	**0.000**	0.005	**0.000**	0.085
**Steg vs Mar**	**0.000**	0.182	**0.000**	0.003	**0.000**	0.043
**Steg vs Orn**	**0.000**	0.706	**0.000**	0.056	**0.000**	0.086
**Bi vs Mar**	0.842	0.564	0.980	0.216	0.017	0.640
**Bi vs Thy**	**0.000**	0.219	**0.000**	0.006	**0.000**	0.153
**Mar vs Thy**	**0.000**	0.175	**0.000**	0.002	**0.000**	0.072
**Orn vs Mar**	0.871	0.143	0.924	0.053	**0.000**	0.456
**Orn vs Thy**	**0.000**	0.409	**0.000**	0.034	**0.000**	0.113

Abbreviations: **HLHAPW**, humerus length against anteroposterior width; **HLHMLW**, humerus length against mediolateral width; **HLHDPCW**, humerus length against deltopectoral crest width; **Ank**, Ankylosauria; **Bi**, basal bipedal ornithischians; **Cer**, Ceratopsidae; **Had**, Hadrosauridae; **Mar**, Marginocephalia; **Orn**, Ornithopoda; **Steg**, Stegosauria; **Thy**, Thyreophora; **p(same)**, the probability that the adjusted means of the two groups differ, cells highlighted in bold are those comparisons that can be statistically distinguished at the p = 0.002 level; **p(HoS)**, the probability that the slopes of the two groups are the same, cells highlighted in bold are those comparisons that can be statistically distinguished at the p = 0.002 level. P = 0.002 is the significance level applied using the Bonferroni correction for multiple pairwise comparisons.

**Table 7:** Pairwise ANCOVAs of the radius.

	RLRAPW		RLRMLW	
Taxon pair	p(same)	p(HoS)	p(same)	p(HoS)
**Ank vs Cer**	0.411	0.726	0.014	0.379
**Ank vs Had**	**0.000**	0.104	**0.000**	**0.001**
**Ank vs Steg**	0.874	0.710	0.040	0.186
**Ank vs Bi**	0.066	0.066	**0.001**	0.051
**Ank vs Mar**	0.334	0.406	0.010	0.120
**Ank vs Orn**	**0.000**	0.456	**0.000**	0.003
**Cer vs Had**	**0.000**	0.010	0.019	0.025
**Cer vs Steg**	0.321	0.603	0.003	0.829
**Cer vs Bi**	0.025	0.046	0.087	0.614
**Cer vs Thy**	0.239	0.540	**0.000**	0.944
**Cer vs Orn**	**0.000**	0.997	0.098	0.033
**Had vs Steg**	**0.000**	0.422	**0.000**	0.307
**Had vs Bi**	0.002	0.361	0.169	0.113
**Had vs Mar**	**0.000**	0.815	0.018	0.146
**Had vs Thy**	**0.000**	0.025	**0.000**	0.006
**Steg vs Bi**	0.466	0.183	0.005	0.859
**Steg vs Mar**	0.868	0.690	0.005	0.821
**Steg vs Orn**	0.000	0.799	**0.000**	0.442
**Bi vs Mar**	0.229	0.550	0.232	0.858
**Bi vs Thy**	0.009	0.008	**0.000**	0.372
**Mar vs Thy**	0.330	0.234	**0.000**	0.325
**Orn vs Mar**	**0.000**	0.561	0.117	0.233
**Orn vs Thy**	**0.000**	0.315	**0.000**	0.018

Abbreviations: **RLRAPW**, radius length against anteroposterior width; **RLRAPW**, radius length against mediolateral width; **Ank**, Ankylosauria; **Bi**, basal bipedal ornithischians; **Cer**, Ceratopsidae; **Had**, Hadrosauridae; **Mar**, Marginocephalia; **Orn**, Ornithopoda; **Steg**, Stegosauria; **Thy**, Thyreophora; **p(same)**, the probability that the adjusted means of the two groups differ, cells highlighted in bold are those comparisons that can be statistically distinguished at the p = 0.002 level; **p(HoS)**, the probability that the slopes of the two groups are the same, cells highlighted in bold are those comparisons that can be statistically distinguished at the p = 0.002 level. P = 0.002 is the significance level applied using the Bonferroni correction for multiple pairwise comparisons.

**Table 8:** Pairwise ANCOVAs of the ulna.

	ULUAPW		ULUMLW	
Taxon pair	p(same)	p(HoS)	p(same)	p(HoS)
**Ank vs Cer**	0.140	0.171	0.007	0.591
**Ank vs Had**	0.005	**0.026**	**0.000**	0.825
**Ank vs Steg**	0.714	0.107	0.327	0.319
**Ank vs Bi**	0.749	0.159	0.054	0.758
**Ank vs Mar**	0.215	0.085	0.007	0.724
**Ank vs Orn**	0.013	**0.019**	**0.000**	0.804
**Cer vs Had**	0.020	0.123	**0.000**	0.065
**Cer vs Steg**	0.173	0.395	0.694	**0.008**
**Cer vs Bi**	0.164	0.847	0.243	0.846
**Cer vs Thy**	0.086	0.997	0.272	**0.003**
**Cer vs Orn**	0.175	0.109	**0.000**	0.072
**Had vs Steg**	**0.000**	0.726	**0.000**	0.398
**Had vs Bi**	0.094	0.082	0.017	0.076
**Had vs Mar**	0.014	0.193	**0.000**	**0.018**
**Had vs Thy**	**0.000**	0.334	**0.000**	0.273
**Steg vs Bi**	0.845	0.290	0.356	0.074
**Steg vs Mar**	0.162	0.520	0.706	**0.012**
**Steg vs Orn**	0.002	0.677	**0.000**	0.538
**Bi vs Mar**	0.310	0.555	0.041	0.666
**Bi vs Thy**	0.759	0.896	0.058	**0.020**
**Mar vs Thy**	0.108	0.672	0.179	**0.003**
**Orn vs Mar**	0.191	0.164	**0.000**	**0.007**
**Orn vs Thy**	**0.000**	0.335	**0.000**	0.431

Abbreviations: **ULUMLW**, ulna length against anteroposterior width; **ULUAPW**, ulna length against mediolateral width; **Ank**, Ankylosauria; **Bi**, basal bipedal ornithischians; **Cer**, Ceratopsidae; **Had**, Hadrosauridae; **Mar**, Marginocephalia; **Orn**, Ornithopoda; **Steg**, Stegosauria; **Thy**, Thyreophora; **p(same)**, the probability that the adjusted means of the two groups differ, cells highlighted in bold are those comparisons that can be statistically distinguished at the p = 0.002 level; **p(HoS)**, the probability that the slopes of the two groups are the same, cells highlighted in bold are those comparisons that can be statistically distinguished at the p = 0.002 level. P = 0.002 is the significance level applied using the Bonferroni correction for multiple pairwise comparisons.

**Table 9:** Pairwise comparisons of radius and humerus length.

	RLHL	
Taxon pair	p(same)	p(HoS)
**Ank vs Cer**	0.044	0.681
**Ank vs Had**	**0.000**	0.863
**Ank vs Steg**	0.255	0.431
**Ank vs Bi**	0.674	0.586
**Ank vs Mar**	0.164	0.568
**Ank vs Orn**	0.007	0.990
**Cer vs Had**	**0.000**	0.811
**Cer vs Steg**	0.009	0.278
**Cer vs Bi**	0.619	0.341
**Cer vs Thy**	0.003	0.311
**Cer vs Orn**	**0.000**	0.771
**Had vs Steg**	**0.000**	0.251
**Had vs Bi**	0.048	0.045
**Had vs Mar**	**0.000**	0.005
**Had vs Thy**	**0.000**	0.288
**Steg vs Bi**	0.317	0.901
**Steg vs Mar**	0.014	0.869
**Steg vs Orn**	0.004	0.605
**Bi vs Mar**	0.770	0.934
**Bi vs Thy**	0.479	0.489
**Mar vs Thy**	0.013	0.466
**Orn vs Mar**	**0.000**	0.004
**Orn vs Thy**	**0.000**	0.698

Abbreviations: **RLHL**, radius length against humerus length; **Ank**, Ankylosauria; **Bi**, basal bipedal ornithischians; **Cer**, Ceratopsidae; **Had**, Hadrosauridae; **Mar**, Marginocephalia; **Orn**, Ornithopoda; **Steg**, Stegosauria; **Thy**, Thyreophora; **p(same)**, the probability that the adjusted means of the two groups differ, cells highlighted in bold are those comparisons that can be statistically distinguished at the p = 0.002 level; **p(HoS)**, the probability that the slopes of the two groups are the same, cells highlighted in bold are those comparisons that can be statistically distinguished at the p = 0.002 level. P = 0.002 is the significance level applied using the Bonferroni correction for multiple pairwise comparisons.

### Femoral Scaling

**Figure 3:**
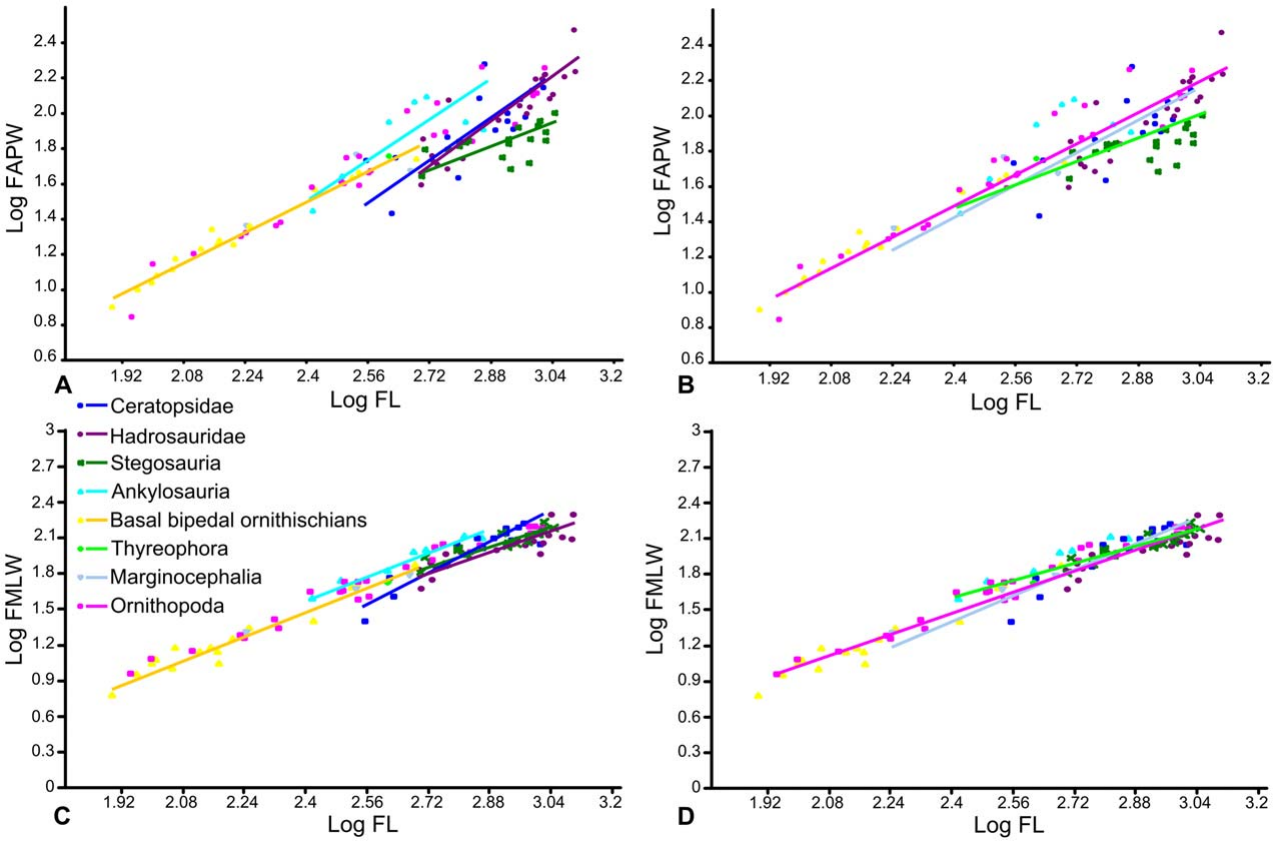
RMA regressions of femur length against width. A–B, femur length against anteroposterior width; C–D, femur length against mediolateral width. A, C, regression lines of Stegosauria, Ankylosauria, Hadrosauridae, Ceratopsidae and basal bipedal ornithischians; B, D, regression lines of Thyreophora, Ornithopoda and Marginocephalia. Abbreviations: **FAPW**, femur anteroposterior width; **FL**, femur length; **FMLW**, femur mediolateral width.

Scaling relationships between femoral length and anteroposterior width, and femoral length and mediolateral width were investigated separately (Fig. 2A, B). An RMA regression of femoral length against anteroposterior width using the entire dataset produced an allometric coefficient (a) of 1.05 (a = 1 indicates isometric scaling). A T-test suggests that this slope does not vary significantly from isometry (p = 0.23). However, there appeared to be discrete clusters in the dataset, so each phylogenetic group was regressed separately (Fig. 3A, B; Table 1). Basal bipedal ornithischians have an allometric coefficient showing very weak positive allometry (a = 1.09; n = 16) that is not statistically significantly different from isometry (p = 0.09). Ankylosaurs and ceratopsids both showed stronger positive allometry (a = 1.46 and 1.54), but could not be statistically distinguished from isometry (p = 0.33 and 0.11 respectively), probably because of relatively small sample sizes (n = 7 and 14 in each case). Stegosaurs showed weak negative allometry (a = 0.82; n = 19), but isometry could not be rejected (p = 0.28). In contrast, hadrosaurs showed strong positive allometry (a = 1.62; n = 21) and isometry could be rejected (p = 0.002). Thyreophora showed the same allometric coefficient as the stegosaurs, probably because the latter formed the largest constituent of the group (a = 0.82; n = 27), but isometry could not be rejected (p = 0.24). Marginocephalia showed weaker positive allometry than Ceratopsidae alone (a = 1.19, n = 17), and isometry could not be rejected. Ornithopoda taken as a whole displayed a similar pattern, with weaker positive allometry than Hadrosauridae alone (a = 1.11) that was statistically distinguishable from isometry (p = 0.01).

**Figure 4:**
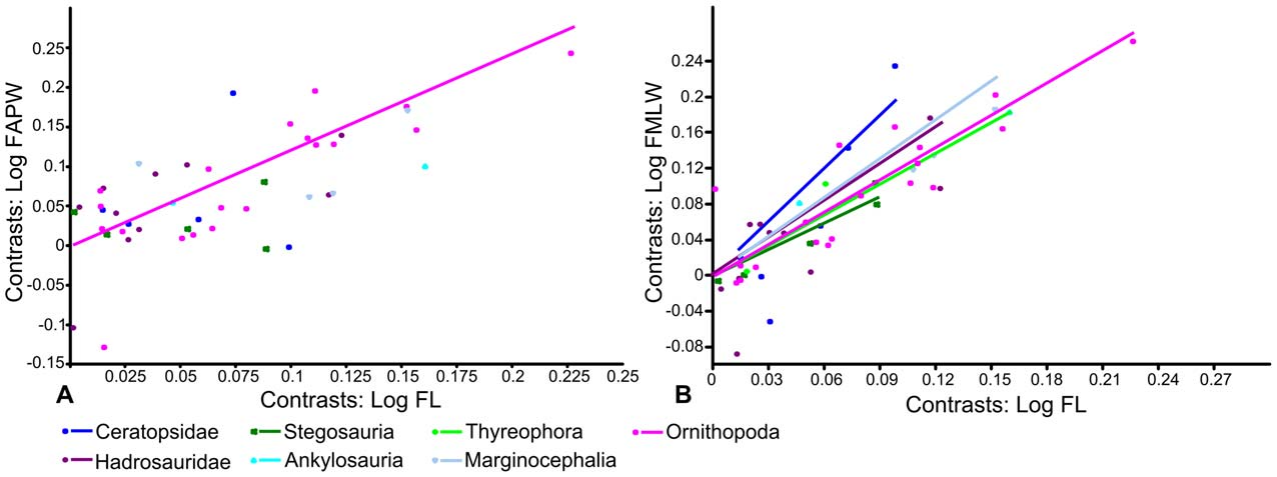
RMA regressions of standardized contrasts of femur length against femur width. A, femur length against anteroposterior width; B, femur length against mediolateral width. Regression lines are forced through the origin. Regression lines for groups in which there was no statistically significant correlation between X and Y at the p = 0.05 level are not shown. Abbreviations: **FAPW**, femur anteroposterior width; **FL**, femur length; **FMLW**, femur mediolateral width.

Using Independent Contrasts (Fig. 4A; Table 1), no correlation between femoral length and anteroposterior width was found in Stegosauria, Hadrosauridae, Ceratopsidae, Thyreophora or Marginocephalia. Only two contrasts were calculated for Ankylosauria, so a regression of the contrasts was not possible. Weak positive allometry was identified when the entire dataset was analysed (a = 1.11, n = 55) this value being slightly higher than that obtained using RMA regression (a = 1.04), but isometry could not be rejected (p = 0.14). Ornithopoda also showed positive allometry (a = 1.21, n = 29), slightly stronger than that obtained using RMA regression (a = 1.11). However, in contrast to the result from RMA regression, isometry could not be rejected (p = 0.06).

Twenty-three pairwise ANCOVAs were carried out to compare the adjusted means and homogeneity of slopes of different groupings. The application of the Bonferroni Correction resulted in a reduction in the significance level at which the null hypothesis could be rejected to 0.0022. At this significance level, the means of only the stegosaurs and hadrosaurs and stegosaurs and ornithopods could be distinguished (Table 5). Examination of the regressions suggests that stegosaurs have a relatively narrower anteroposterior width for a given femoral length. Hadrosaurs and stegosaurs also had significantly different slopes, as did stegosaurs and basal bipedal ornithischians, hadrosaurs and thyreophorans, basal bipedal ornithischians and thyreophorans, and ornithopods and thyreophorans.

When femoral length and mediolateral width were regressed for the entire dataset (n = 107), slight, statistically significant positive allometry was found (a = 1.15; p = 0.00). When each phylogenetic grouping was regressed separately (Fig. 3C, D; Table 1), ankylosaurs and hadrosaurs showed positive allometry (a = 1.21 and 1.12 respectively) but this could not be distinguished from isometry (p = 0.11 and 0.37 respectively). Basal bipedal ornithischians (a = 1.30; p = 0.004), ceratopsids (a = 1.67; p = 0.005) and Marginocephalia (a = 1.37; p = 0.008) showed relatively strong positive allometry that was significantly different from isometry. Ornithopods (including hadrosaurs) showed weak positive allometry (a = 1.12) that was significantly different from isometry (p = 0.001). In contrast, stegosaurs and thyreophorans showed slight negative allometry (a = 0.976 and 0.912, respectively) but this could not be distinguished from isometry (p = 0.804 and 0.238, respectively). Using Independent Contrasts (Fig. 4B; Table 1), femoral length and mediolateral width were correlated significantly in all groups examined, except for Ankylosauria, in which only two contrasts were computed. The allometric coefficients were slightly increased for all groups, but only Ornithischia (the entire dataset) and Ornithopoda could be statistically differentiated from isometry, probably due to reduced sample sizes. Thyreophora showed weak, non-significant positive allometry (a = 1.15; p = 0.159), in contrast to the slight negative allometry observed using RMA regression.

Pairwise ANCOVAs using the Bonferroni Correction showed that the mean of the ankylosaur group was different to all other groups except basal bipedal ornithischians (Table 5). Examination of the regressions indicates that ankylosaurs have a greater mediolateral width for a given femoral length than other groups. Hadrosaurs could also be distinguished from thyreophorans. Homogeneity of slope suggested significant differences only between ceratopsids and thyreophorans, basal bipedal ornithischians and thyreophorans, and marginocephalians and thyreophorans.

### Humeral scaling

**Figure 5:**
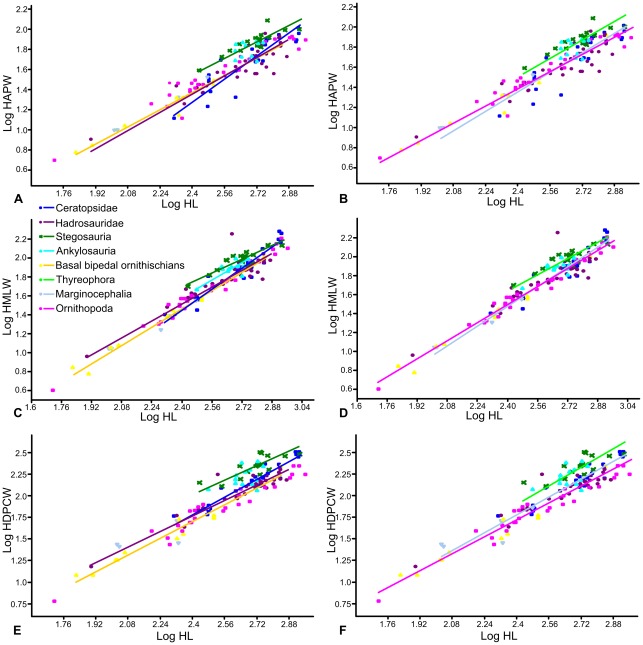
RMA regressions of humerus length against width. A–B, humerus length against anteroposterior width; C–D, humerus length against mediolateral width; E–F, humerus length against deltopectoral crest width. A, C, E, regression lines of Stegosauria, Ankylosauria, Hadrosauridae, Ceratopsidae and basal bipedal ornithischians; B, D, F regression lines of Thyreophora, Ornithopoda and Marginocephalia. Regression lines for groups in which there was no statistically significant correlation between X and Y at the p = 0.05 level are not shown. Abbreviations: **HAPW**, humerus anteroposterior width; **HDPCW**, humerus width across the deltopectoral crest; **HL**, humerus length; **HMLW**, humerus mediolateral width.

Humerus length was regressed against minimum mediolateral width, anteroposterior width at the same location on the humeral shaft as the former measurement, and maximum width across the deltopectoral crest (Fig. 2C, D). Humerus length against anteroposterior width for the entire dataset showed weak positive allometry that is significantly distinguished from isometry (a = 1.17; p<0.00). No correlation between humeral length and anteroposterior width was identified in ankylosaurs (probability that X and Y are uncorrelated  = 0.56) so they will not be considered further. Only the ceratopsid (1.44, p = 0.005) and marginocephalian (a = 1.21, p = 0.02) datasets could be statistically differentiated from isometry (Fig. 5A, B; Table 2).

**Figure 6:**
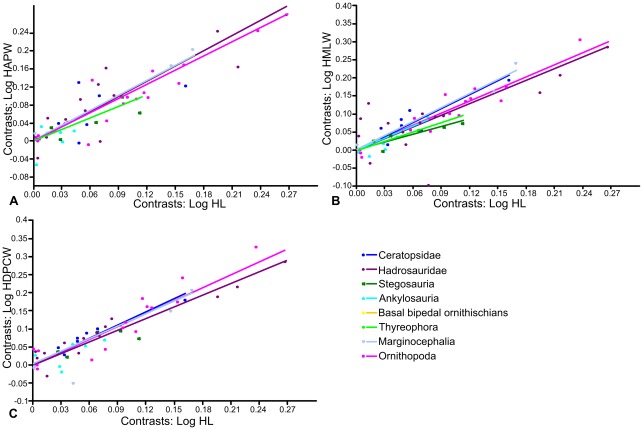
RMA regressions of standardized contrasts of humerus length against humerus width. A, humerus length against anteroposterior width; B, humerus length against mediolateral width; C, humerus length against deltopectoral crest width. Regression lines are forced through the origin. Regression lines for groups in which there was no statistically significant correlation between X and Y at the p = 0.05 level are not shown. Abbreviations: **HAPW**, humerus anteroposterior width; **HDPCW**, humerus width across the deltopectoral crest; **HL**, humerus length; **HMLW**, humerus mediolateral width.

Using Independent Contrasts (Fig. 6A; Table 2), no correlation between humeral length and anteroposterior width was found in Ankylosauria, Stegosauria and Ceratopsidae, probably due to small sample sizes (n = 6, 6 and 7 respectively). The allometric coefficients of the entire dataset and Marginocephalia reduced slightly, becoming statistically indistinguishable from isometry. The allometric coefficients in Hadrosauridae and Ornithopoda were very similar to those obtained using RMA regression and could not be distinguished from isometry. The allometric coefficient for Thyreophora changed from weak positive allometry using RMA regression to weak negative allometry (a = 0.82) using Independent Contrasts, but neither was significantly different from isometry. Pairwise ANCOVAs indicated that the mean of the stegosaur dataset was significantly different from all other groups except ankylosaurs. The mean of the ankylosaur dataset could be statistically distinguished from the mean of the hadrosaur and ornithopod dataset and the mean of the thyreophoran dataset could be statistically distinguished from all others (Table 6). Examination of the regressions suggests that thyreophorans have a relatively greater anteroposterior width for a given femoral length than the other groups. None of the slopes could be statistically distinguished from each other.

RMA regression of humerus length against mediolateral width for the entire dataset suggested positive allometry that could be significantly distinguished from isometry (a = 1.27; p>0.000). When the phylogenetic groups were regressed separately (Fig. 5C, D; Table 2), basal bipedal ornithischians (a = 1.19), ornithopods (a = 1.19) and hadrosaurs (a = 1.12) showed weak positive allometry that was statistically significant in ornithopods (p = 0.000) and basal bipedal ornithischians (p = 0.003) but not in hadrosaurs (p = 0.178). Stegosaurs showed weak negative allometry (a = 0.92) while ankylosaurs (a = 1.15) and thyreophorans (a = 1.06) showed weak positive allometry, but none could be statistically differentiated from isometry. Ceratopsids (a = 1.41) and marginocephalians (a = 1.32) showed stronger positive allometry than observed in any of the other groups, and in both cases isometry could be rejected (p = 0.000). Using Independent Contrasts (Fig. 6B; Table 2), the allometric coefficient decreased slightly in all cases but generally showed the same trends. Ceratopsids (a = 1.28; p = 0.08) and ornithopods (a = 0.12; p = 0.15) could no longer be distinguished from isometry. Stegosaurs showed strong negative allometry (a = 0.69) statistically distinguishable from isometry (p = 0.01). Pairwise ANCOVAs (Table 6) suggested that the means of the stegosaur and thyreophoran datasets could be statistically distinguished from all other groups, and the ankylosaurs could be distinguished from the ornithopods. The slopes of the ceratopsids and thyreophoran datasets could be distinguished from each other, as could the slopes of the marginocephalian and thyreophoran datasets.

Humerus length plotted against width across the deltopectoral crest indicated positive allometry for the entire dataset (1.36; p<0.000). No correlation between humeral length and width across the deltopectoral crest was identified in ankylosaurs (probability that X and Y are uncorrelated  = 0.21). Stegosaurs (a = 1.07) showed weak positive allometry while that of thyreophorans (a = 1.32) was stronger, but in neither case could isometry be rejected (p = 0.71 and 0.1, respectively). Hadrosaurs also showed weak positive allometry statistically indistinguishable from isometry (a = 1.13, p = 0.06). Basal bipedal ornithischians (a = 1.21, p = 0.03) and ornithopods (a = 1.22, p = 0.00) showed positive allometry significantly different from isometry. Once again, ceratopsids (a = 1.36, p = 0.00) and marginocephalians (a = 1.28, p = 0.00) showed statistically significant positive allometry (Fig. 5E, F; Table 2).

Using Independent Contrasts, humerus length and deltopectoral crest width did not correlate in Ankylosauria, Stegosauria or Thyreophora. In the other groups, allometric coefficients were decreased relative to those using RMA regression, but overall patterns were similar (Fig. 6C; Table 2). Hadrosaurs showed weak positive allometry not distinguishable from isometry (a = 1.08, p = 0.15), while ornithopods showed weak positive allometry that could be statistically differentiated from isometry (a = 1.19, p = 0.001). Ceratopsids showed statistically significant positive allometry (a = 1.22, a = 0.03), but that of marginocephalians was no longer statistically significant (a = 1.17, p = 0.15). Pairwise ANCOVAs (Table 6) showed that the mean of the stegosaur group was statistically distinguishable from the means of all other groups except ankylosaurs. Ankylosaurs were statistically different from hadrosaurs, bipedal basal ornithischians and ornithopods, while the mean of the thyreophoran dataset was once again statistically distinguishable from all other groups. Examination of the regressions suggests that thyreophorans have a greater deltopectoral crest width than the other groups for a given humeral length. The means of the ceratopsid and marginocephalian groups could be statistically distinguished from that of the ornithopods, the latter appearing to have a smaller deltopectoral crest width for a given humeral length. No statistically different slopes were found.

### Radial scaling

**Figure 7:**
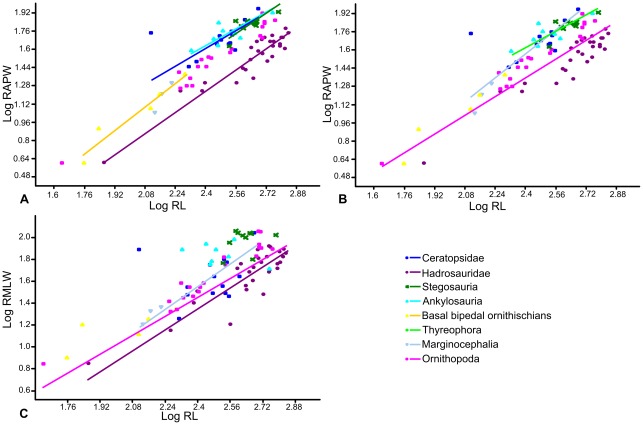
RMA regressions of radius length against width. A–B, radius length against anteroposterior width; A, regression lines of Stegosauria, Ankylosauria, Hadrosauridae, Ceratopsidae and basal bipedal ornithischians; B, regression lines of Thyreophora, Ornithopoda and Marginocephalia. C, radius length against mediolateral width. Regression lines for groups in which there was no statistically significant correlation between X and Y at the p = 0.05 level are not shown. Abbreviations: **RAPW**, radius anteroposterior width; **RL**, radius length; **RMLW**, radius mediolateral width.

Radial length was regressed against midshaft anteroposterior width and mediolateral width separately (Fig. 2G, H). Radius length against anteroposterior width for the entire dataset showed statistically significant weak positive allometry (a = 1.14; p = 0.05). When analyzed separately (Fig. 7A, B; Table 3), ankylosaurs (a = 0.85), ceratopsids (a = 0.98) and thyreophorans (a = 0.85) showed weak negative allometry, while stegosaurs (a = 1.06), bipedal basal ornithischians (a = 1.32), ornithopods (a = 1.04) and marginocephalians (a = 1.34) showed positive allometry, but none could be statistically differentiated from isometry. Hadrosaurs showed weak positive allometry significantly different from isometry (a = 1.16, p = 0.05).

**Figure 8:**
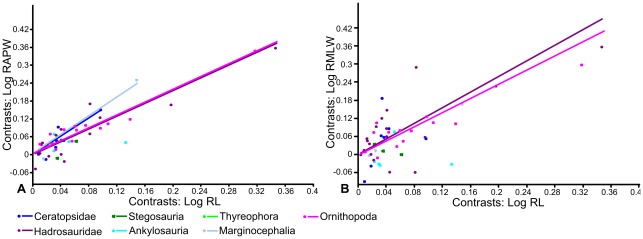
RMA regressions of standardized contrasts of radius length against radius width. A, radius length against anteroposterior width; B, radius length against mediolateral width. Regression lines are forced through the origin. Regression lines for groups in which there was no statistically significant correlation between X and Y at the p = 0.05 level are not shown. Abbreviations: **RAPW**, radius anteroposterior width; **RL**, radius length; **RMLW**, radius mediolateral width.

Independent Contrasts (Fig. 8A; Table 3) revealed no correlation between radius length and anteroposterior width for ankylosaurs and thyreophorans, and only two contrasts could be computed for stegosaurs, so regressions could not be carried out. The remaining groups had allometric coefficients similar to those derived from RMA regressions. The allometric coefficient of the marginocephalian group increased to show strong positive allometry (a = 1.6) significantly different from isometry (p = 0.002). In contrast, the allometric coefficient of hadrosaurs decreased (a = 1.08) and became indistinguishable from isometry (p = 0.38). None of the slopes could be statistically distinguished from each other. Pairwise ANCOVAs (Table 7) showed the adjusted means of the hadrosaur and ornithopod datasets could be significantly distinguished from the other groupings. Examination of the regressions suggests that hadrosaur radii have a narrower anteroposterior width for a given length than the other groups, including basal bipedal ornithischians.

RMA regression of radius length to mediolateral width of the entire dataset revealed weak positive allometry that was statistically significant (a = 1.16, p = 0.05). No correlation between radius length and mediolateral width was identified in ankylosaurs, stegosaurs, ceratopsids, basal bipedal ornithischians or thyreophorans. Hadrosaurs (a = 1.22, p = 0.07), ornithopods (a = 1.09, p = 0.24) and marginocephalians (a = 1.31, p = 0.31) were positively allometric, but this could not be distinguished from isometry (Fig. 7C). Using Independent Contrasts (Fig. 8B; Table 3), there was no correlation between radius length and radius mediolateral width in ankylosaurs, stegosaurs, ceratopsids, thyreophorans and marginocephalians. Hadrosaurs (a = 1.3, p = 0.11) and ornithopods (a = 1.17, p = 0.12) both displayed positive allometry that was not significantly different from isometry. Pairwise ANCOVAs (Table 7) suggested that there was a difference between the adjusted means of the thyreophorans and most of the other groups. Examination suggests that the radius of thyreophorans had a significantly wider mediolateral width for a given radial length than the other groups.

### Ulnar Scaling

Ulna length was regressed against anteroposterior width level with the anterolateral process, and mediolateral width across the medial process (Fig. 2E, F). In the stegosaurian and ankylosaurian datasets, no correlation was found between length and either of the width measurements, and their results were disregarded.

**Figure 9:**
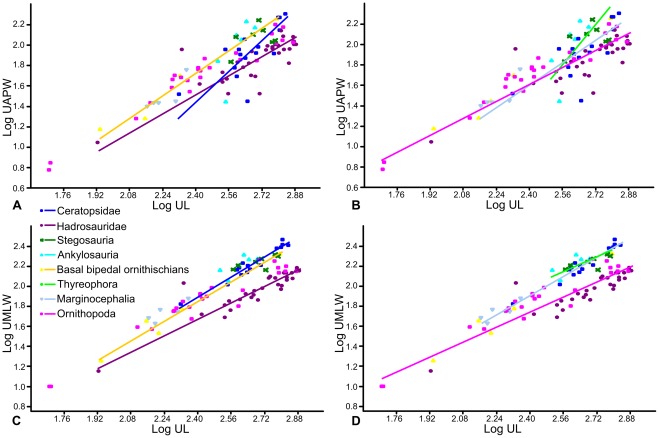
RMA regressions of ulna length against width. A–B, ulna length against anteroposterior width; C–D, ulna length against mediolateral width. A, C, regression lines of Stegosauria, Ankylosauria, Hadrosauridae, Ceratopsidae and basal bipedal ornithischians; B, D regression lines of Thyreophora, Ornithopoda and Marginocephalia. Regression lines for groups in which there was no statistically significant correlation between X and Y at the p = 0.05 level are not shown. Abbreviations: **UAPW**, ulna anteroposterior width; **UL**, ulna length; **UMLW**, ulna mediolateral width.

When ulna length was regressed against anteroposterior width using the entire dataset, weak, statistically significant positive allometry was found (a = 1.17, p = 0.008). Thyreophora showed strong, statistically significant positive allometry (a = 2.49; p = 0.04), while ceratopsids (a = 1.9; p = 0.02) and marginocephalians (a = 1.36, p = 0.039) also showed statistically significant positive allometry, although it was not as strongly positive as in Thyreophora. Hadrosaurs (a = 1.12, p = 0.35), bipedal basal ornithischians (a = 1.35, p = 0.09) and ornithopods (a = 1.05, p = 0.43) showed positive allometry that could not be statistically differentiated from isometry (Fig. 9A, B; Table 4).

**Figure 10:**
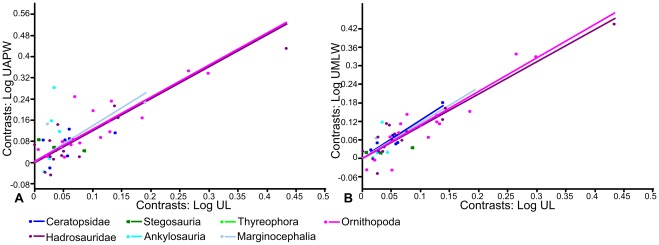
RMA regressions of standardized contrasts of ulna length against ulna width. A, ulna length against anteroposterior width; B, ulna length against mediolateral width. Regression lines are forced through the origin. Regression lines for groups in which there was no statistically significant correlation between X and Y at the p = 0.05 level are not shown. Abbreviations: **UAPW**, ulna anteroposterior width; **UL**, ulna length; **UMLW**, ulna mediolateral width.

Using Independent Contrasts (Fig. 10A; Table 4), no correlation was found between ulna length and anteroposterior width in Ankylosauria, Stegosauria, Ceratopsidae or Thyreophora. In the other groups the allometric coefficient increased in comparison with the results from the RMA regressions. Hadrosaurs showed positive allometry that was not distinguishable from isometry (a = 1.2, p = 0.16) as they did using RMA regression. The allometric coefficient of Ornithopoda increased to become statistically significantly different from isometry (a = 1.2, p = 0.01), while that of Marginocephalia remained the same, but became non-significant (a = 1.36, p = 0.18). No slopes were found to be statistically different from each other. Pairwise ANCOVAs (Table 8) suggested that the mean of the hadrosaur dataset could be statistically distinguished from that of the stegosaurs, and the mean of the ornithopod dataset could be distinguished from that of the thyreophorans. Examination of the regressions showed that stegosaurs had more robust ulnae in an anteroposterior orientation for a given ulna length.

Regression of ulna length against ulna mediolateral width for the entire dataset revealed weak positive allometry not distinguishable from isometry (a = 1.1; p = 0.07), and a similar result was obtained for basal bipedal ornithischians (a = 1.24; p = 0.16). Hadrosaur ulnae were found to scale isometrically (a = 1.02; p = 0.81), Thyreophora (a = 0.88; p = 0.55) and Ornithopoda (a = 0.95; p = 0.32) scaled with weak negative allometry not significantly distinguishable from isometry, while Ceratopsidae (a = 1.29; p = 0.005) and Marginocephalia (a = 1.19; p = 0.001) scaled with weak positive allometry statistically differentiated from isometry (Fig. 9C, D; Table 4).

When phylogeny was taken into account using Independent Contrasts (Fig. 10B; Table 4), no relationship was found between X and Y for Ankylosauria, Stegosauria or Thyreophora. The allometric coefficient for Ornithopoda increased to show weak positive allometry not distinguishable for isometry (a = 1.08; p = 0.13), while that of Marginocephalia decreased to become indistinguishable from isometry (a = 1.16; p = 0.15). The allometric coefficient of Ceratopsidae remained weakly positive and significantly different from isometry (a = 1.23; p = 0.05). Pairwise comparisons of adjusted group means using ANCOVAs (Table 8) revealed that hadrosaurs and ornithopods were statistically different from all other groups except basal bipedal ornithischians. Hadrosaur ulnae were found to be significantly narrower mediolaterally than in the other groups.

### Radius/humerus ratio

Hadrosaurs have the highest radius/humerus ratios (mean  = 1.03), while ceratopsids have the lowest ratios (mean  = 0.60). Ankylosaurs (mean  = 0.66) and stegosaurs (mean  = 0.68) have values similar to ceratopsids. Basal bipedal ornithischians also have relatively low ratios (mean  = 0.64). Pairwise ANCOVAs of radius length against humerus length (Table 9) show that the adjusted means of hadrosaurs can be statistically distinguished from all other groups except basal bipedal ornithischians; ornithopods can also be distinguished from all other groups except ankylosaurs and stegosaurs.

### Cluster analysis

**Figure 11:**
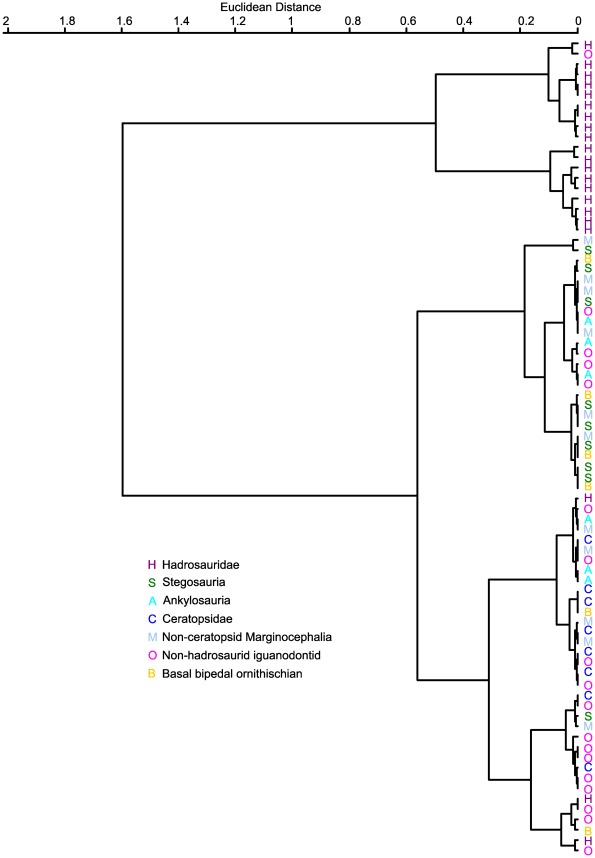
Ward '**s Cluster Analysis dendrogram.** The length of the branches is the Euclidian distance, the distance between two points in multidimensional space.

Cluster analyses were carried out on the radius/humerus ratio. Ward's cluster analysis produced two major clusters (Figure 11). One major cluster contained only hadrosaurs and the non-hadrosaurid hadrosauriform *Bactrosaurus*. All hadrosaurs in the sample were present in this cluster except for an indeterminate hadrosaurid (CMN 40603), an individual of *Hypacrosaurus altispinus* (USNM 11590) and an individual of *Gryposaurus notabilis* (TMP 1980.22.01). The other major cluster contained all other ornithischians, which were not further differentiated into clusters representing individual phylogenetic groups.

K-means cluster analysis was carried out five times (SI). Iterations one and two produced the same results, and iterations three, four and five produced the same results, which differed slightly from those of one and two. However, in each iteration, basal ornithischians, thyreophorans, marginocephalians and non-hadrosaurid ornithopods were represented by two groups, and hadrosaurs were represented by two different groups. In iterations one and two, the hadrosaurs that clustered with non-hadrosaurid taxa in the Ward's analysis also clustered with these taxa in the K-means analysis. The basal ceratopsian *Yinlong downsi* (IVPP V14530) and the ceratopsid *Vagaceratops irvinensis* (CMN 41357) clustered with the hadrosaurs. In iterations three and four, *Hypacrosaurus altispinus* (USNM 11950) and *Maiasaura peeblesorum* (ROM 44771) clustered with the basal ornithischians, thyreophorans, marginocephalians and non-hadrosaurid ornithopods, while *Gasparinisaura cincolsaltensis* (NMST 20392) clustered with the hadrosaurs. Within hadrosaurs, no clusters were found to represent the two major hadrosaur clades (Saurolophinae and Lambeosaurinae), suggesting that radius/humerus ratios do not differ consistently between them.

## Discussion

### Are differences in femoral scaling observable between bipedal and quadrupedal ornithischians

When femoral length was regressed against femoral anteroposterior width, hadrosaurs were found to display strong positive allometry (a = 1.62) statistically distinguishable from isometry. The addition of non-hadrosaurid ornithopods into the dataset reduced the allometric coefficient to 1.11, showing that hadrosaurs scale differently to ornithopods as a whole. Allometry in the other groups was statistically indistinguishable from isometry. Stegosaurs and hadrosaurs had statistically different allometric coefficients, probably because stegosaurs had the lowest allometric coefficient, while hadrosaurs had the highest. When femoral length and femoral mediolateral width were examined, ceratopsids were found to display strong positive allometry (a = 1.67, increasing to a = 1.99 if phylogeny is taken into account using Independent Contrasts) significantly different from isometry. The addition of non-ceratopsid marginocephalians into the dataset produced weaker positive allometry (a = 1.37). Basal bipedal ornithischians and ornithopods also displayed positive allometry but it was weaker than that of ceratopsids and marginocephalians. Allometry in other groups could not be distinguished from isometry, but the allometric coefficient of thyreophorans and basal bipedal ornithischians, marginocephalians and ceratopsids could be distinguished statistically, probably because thyreophorans had the lowest allometric coefficient.

These results suggest that as femoral length increases in hadrosaurs, the anteroposterior width increases rapidly, but the mediolateral width increases more slowly. This produces femoral eccentricity of the midshaft in large hadrosaurs such that the anteroposterior dimension is larger than the mediolateral dimension. This pattern is not observed in ornithopods as a whole, where both width measurements increase at the same rate. This provides evidence for differences in gait or hind limb use between hadrosaurs and other ornithopods. The largest hadrosaurs, such as *Shantungosaurus* [16] were not included in this study, and the addition of these taxa would be useful to confirm whether the trend continues in the largest of hadrosaurs. In contrast, the opposite pattern is observed in ceratopsids, where mediolateral width increases rapidly with increasing femoral length, while anteroposterior width increases more slowly, producing midshaft eccentricity where the mediolateral dimension is larger than the anteroposterior dimension in large femora. Marginocephalians as a total group also display an increase in mediolateral width more rapidly than anteroposterior width as femoral length increases; however, this is not as marked as in ceratopsids.

Midshaft eccentricity of the femur, where the mediolateral dimension is greater than the anteroposterior dimension, has previously been identified in many large dinosaurs [15], [79]. In sauropods, this eccentricity is correlated with a wide-gauged stance [80]. The ground reaction force acts upwards from the foot to the center of mass during locomotion. For an animal that places its feet on the midline during locomotion, the main vector of the ground reaction force is nearly vertical, because the foot is placed directly underneath the center of mass. Since the long axis of the femur is held at an oblique angle to vertical (the hind limb is flexed), the largest stresses on the femur will therefore be directed in an anteroposterior orientation. In contrast, for animals that place their feet lateral to the midline during locomotion, the ground reaction force will contain a greater transverse vector, and stress in the mediolateral direction will be increased [80]. Although direct measurements of avian femoral strain have shown torsional stresses to be high [81], bird locomotion is somewhat different to that of non-avian dinosaurs because birds use a rotation-based method of lateral limb support [82] rather than the abduction-based mode used in dinosaurs, including ornithischians [83]. The avian femur is also held more horizontally [81], [84]. Reorientation and increased rotation of the femur in birds is thought to have increased torsional stresses relative to those of dinosaurs [84], so measures of peak locomotor strains in birds may not be representative of the condition in non-avian dinosaurs.

It is therefore possible that the difference in midshaft eccentricity with increasing femoral size in hadrosaurs and ceratopsids results from a difference in stance and gait. Trackways indicate that hadrosaurs placed their feet on the midline during locomotion [12] in a ‘narrow-gauged’ stance. Ceratopsid trackways are rare in the fossil record [85], but consideration of how the reaction forces might affect femoral morphology suggests that midshaft eccentricity indicates a wider-gauged stance than in hadrosaurs.

Basal bipedal ornithischians also appear to show slight positive allometry in mediolateral width but not in anteroposterior width. Positive allometry in the mediolateral direction is much lower than it is in ceratopsids, and all basal bipedal taxa are much smaller than the ceratopsid taxa. Their femora are also generally anteroposteriorly bowed along their length, unlike in larger taxa such as ceratopsids, which tend to have columnar femora. Midshaft eccentricity in a mediolateral orientation in combination with anteroposterior bowing is observed in the long bones of many taxa, and has not been satisfactorily explained from a biomechanical perspective [86]. One hypothesis suggests that the combination of anteroposterior bowing and midshaft eccentricity in a long bone acts to generate strain, and that this is necessary because intermittent straining to a certain threshold acts to confer some as yet unidentified benefit to bone tissue [86]. This hypothesis is unlikely to explain the midshaft eccentricity observed in larger ceratopsids because their femora are not bowed along their length. It seems likely that different selective pressures acted to produce midshaft eccentricity in basal bipedal ornithischians and ceratopsids.

Thyreophorans appear to have differently shaped femora from the neornithischian groups. Pairwise ANCOVAs suggested that stegosaurs had more slender femora in an anteroposterior direction for a given femoral length than hadrosaurs and ornithopods, while ankylosaurs had wider femora in a mediolateral direction for a given femoral length than the other groups. These differences could be interpreted in the light of differences in body mass, though confirmation of this hypothesis requires the production of consensual body mass estimates for these taxa. Stegosaurs may have had a lower body mass than hadrosaurs with the same femoral length, while ankylosaurs may have had a greater body mass than other ornithischians with the same femoral length. RMA regression analyses and Independent Contrasts reveal that thyreophoran allometry of the femur cannot be distinguished from isometry. This suggests thyreophoran femora do not display the same scaling differences related to stance and locomotion as ceratopsids and hadrosaurs. This might be partially due to small sample sizes and a narrow species sample. The addition of more taxa would further illuminate scaling relationships in thyreophorans and their relationships to stance, locomotion and body size.

We hypothesized that femoral scaling would be similar in quadrupedal and bipedal ornithischians. However, there are clear differences in femoral scaling between bipeds and quadrupeds, and between different quadrupedal clades. Femoral scaling appears to indicate differences between stance and gait beyond the simple division between bipedality and quadrupedality, suggesting that different quadrupedal clades used the hind limb in different ways for both support and locomotion.

### Do differences exist in the humeral scaling of bipedal and quadrupedal ornithischians

When humeral length was regressed against all three width measurements, ceratopsids showed strong positive allometry. Marginocephalia also showed strong positive allometry, although in each case it was reduced relative to Ceratopsidae alone. Other groups showed much weaker positive allometry or allometry that could not be differentiated from isometry in all width orientations, with the exception of Stegosauria. When humeral length was regressed against mediolateral width, stegosaurs showed strong negative allometry that was distinguishable from isometry when phylogeny was taken into account using Independent Contrasts (a = 0.69). Pairwise ANCOVAs found statistically distinguishable slope differences between thyreophorans and marginocephalians and thyreophorans and ceratopsids, although stegosaurs alone were not found to be statistically distinguishable from these groups after application of the Bonferroni Correction. These results falsify our original hypothesis that the humeri of all quadrupedal ornithischians would scale similarly.

Allometric scaling in ceratopsids suggests that as humerus length increases, width increases rapidly to produce more robust humeri in the largest ceratopsids than in smaller members of the clade. The very robust humeri of the largest ceratopsids could be interpreted as an indication that the center of mass was further forward in larger taxa than in smaller taxa, potentially as the result of the proportionally larger head. Investigations of ceratopsian head scaling with respect to body mass suggest that in contrast to the condition in most other tetrapods, the size of the head scales with positive allometry, and that the largest ceratopsians had proportionally larger heads than smaller ceratopsians [87]. Indeed, the largest ceratopsians possessed skulls that reached lengths of up to 50% of trunk length [87].

In contrast, negative allometry in the stegosaur humerus suggests that humerus mediolateral width increases more slowly than humerus length, so that the largest stegosaurs have relatively slender humeri in comparison with the smallest members of the clade. This could be due to changes in the center of mass related to distribution of dermal armor; for example, *Kentrosaurus*, the smallest stegosaur in the sample, is known to have possessed parascapular spines [88]. Despite the large number of individuals of *Stegosaurus* known, no parascapular spine belonging to the genus has ever been discovered [70]. The additional mass of these large dermal spines in the shoulder region might have caused the center of mass to be located further anteriorly in *Kentrosaurus* than in *Stegosaurus*. However, it is not known whether the largest stegosaur, *Dacentrurus*, possessed parascapular spines, nor is the distribution or characteristics of its dermal armor known [89], so this hypothesis cannot be tested further at the current time. Calculations of the center of mass of *Kentrosaurus* and *Stegosaurus* could be used to investigate these differences.

Allometric coefficients of Hadrosauridae, Ornithopoda and basal bipedal ornithischians all indicate weak positive allometry that could not be distinguished from isometry. This could be interpreted as evidence to suggest that hadrosaurs and indeed all ornithopods were bipedal, as has been previously suggested (e.g., [9], [10]). However, morphological ([13], [14]; Methods, above) and trackway [12] evidence suggests that large ornithopods and hadrosaurs walked quadrupedally at least some of the time: consequently, their forelimb elements would need to be adapted to this behaviour to some extent, even if they were not obligate quadrupeds. Clear humeral scaling differences between Ceratopsidae and Stegosauria indicate that there is no scaling relationship that correlates with quadrupedality in ornithischians. A decrease in allometric coefficient when basal marginocephalians are added to the ceratopsid group, and an increase in allometric coefficient when other thyreophorans are added to the stegosaur group to close to isometry in both cases indicate that isometry may be the plesiomorphic condition for Ornithischia. We therefore prefer to interpret isometric scaling of the humerus in hadrosaurs as one of several different scaling patterns seen in ornithischians that use quadrupedality as their predominant form of locomotion, rather than as an indicator of bipedality; however we acknowledge that alternative interpretations are possible.

Pairwise ANCOVAs of humeral length against all three width measurements showed that the means of the stegosaur and thyreophoran groups could be statistically differentiated from all other groups, and that stegosaur and thyreophoran humeri were more robust for a given humerus length than in the other groups. This contrasts with the results for the femur, in which stegosaurs were found to have more slender femora in an anteroposterior orientation than hadrosaurs. The robustness of thyreophoran and particularly stegosaur humeri could be interpreted to indicate that the center of mass of stegosaurs was located more anteriorly than in other ornithischians, so that the humerus was required to support proportionally more of the body mass. However, Henderson [26] modelled the center of mass of several dinosaurs and showed that the center of mass in *Stegosaurus* was located as far posteriorly as it was in bipedal taxa such as *Tyrannosaurus*. Alternatively, the robustness of stegosaur humeri could be related to a specific behaviour. For example, it has been suggested that stegosaurs utilized a tripodal stance [90], [91]; perhaps increased stress on the humerus was generated during rearing as a result of pushing off from the ground.

We hypothesized that bipedal and quadrupedal ornithischians would have different humeral scaling patterns related to the weight-bearing function of the humerus in quadrupeds. We find, however, that no scaling relationship indicates quadrupedality, and that different quadrupedal groups show different scaling patterns. These patterns may relate to clade-specific behaviours or differences in the distribution of the center of mass as the result of the bizarre display structures possessed by the different quadrupedal ornithischian clades.

### Cursorial morphology and locomotor performance in quadrupedal ornithischians

Although the results of RMA regressions and Independent Contrasts are largely inconclusive and sometimes contradictory, pairwise ANCOVAs of adjusted group means suggested that hadrosaurs and ornithopods had more slender radii in an anteroposterior orientation than any other ornithischian group, and that hadrosaurs and ornithopods had more slender ulnae in a mediolateral orientation than other ornithischian groups, with the exception of basal bipedal ornithischians. This provides some evidence to suggest that the forelimb epipodials of ornithopods, including hadrosaurs, were more slender than those of other ornithischians.

Cluster analyses of radius/humerus ratio repeatedly separated hadrosaurs, along with the non-hadrosaurid hadrosauriform *Bactrosaurus johnsoni*, from the other groups. In contrast, marginocephalians, thyreophorans, basal bipedal ornithischians and non-hadrosaurid ornithopods could not be consistently distinguished from each other in the cluster analyses. Pairwise ANCOVAs of radius length and humerus length suggested that the mean of the hadrosaur group could be distinguished from all other groups, and that the mean of the ornithopod group could be distinguished from Thyreophora, Marginocephalia and Ceratopsidae, but not from Ankylosauria and Stegosauria. Hadrosaurs were found to have much higher radius/humerus ratios than any of the other quadrupedal groups examined, and are the only group in which the radius and humerus are approximately the same length. The hadrosaur humerus/radius ratio statistically distinguished hadrosaurs from other ornithischian groups consistently, suggesting that the forelimb epipodials of hadrosaurs are significantly longer than those of other ornithischians.

The elongate and slender epipodials of hadrosaurs might indicate that they display more cursorial morphologies in the forelimb than other quadrupedal ornithischians. This is consistent with other aspects of their locomotor morphology, such as a digitigrade and tightly appressed manus [37]. Cursorial morphologies are correlated with locomotor performance in extant taxa [33], [35]. Our findings suggest that hadrosaurs had a higher locomotor performance than other quadrupedal ornithischians. This could relate to an ability to run faster, but could also relate to more energetically efficient low speed locomotion [35]. Differences in locomotor performance between different quadrupedal ornithischian groups might reflect behavioural differences between clades. For example, increased locomotor performance might indicate long-range migration in hadrosaurs, that hadrosaurs had larger home ranges, or that hadrosaurs used high-speed locomotion as a predominant method of predator escape, whereas other ornithischians relied on display structures such as horns, frills and dermal armor to deter predators. Such behavioural differences cannot be distinguished from limb bone scaling alone. Alternatively, the slender epipodials could indicate that the forelimbs of hadrosaurs were less heavily modified for weight-bearing than in other quadrupedal groups, although this does not negate their ability to walk quadrupedally (see above). In either case, this highlights the diversity of form seen in the limb elements of those clades that adopted quadrupedal habits, and emphasizes the potential differences in gait and stance that existed between them.

### Conclusion

Limb bone scaling is informative about stance and gait in dinosaurs because bones are shaped by the forces acting upon them [86]. The results of our analysis, based on the most complete database of ornithischian limb measurements compiled to date, show significant phylogenetic differences between quadrupedal ornithischian clades. Combining quadrupedal ornithischians into a single functional group can therefore obscure important trends that would otherwise shed light on ornithischian functional morphology and evolution. Ornithischian dinosaurs clearly experimented with locomotor modes, and despite convergence in limb morphology and the constraints imposed by bipedal ancestry, quadrupedal ornithischians appear to have utilized a previously unrealised diversity in stance, gait, and possibly behaviour. Femoral scaling reveals differences in gait between hadrosaurs and ceratopsids, while humeral scaling might be informative about changes in the center of mass related to the evolution of large frills in ceratopsids. Thyreophorans apparently possessed differently proportioned humeri and femora than ceratopsids and ornithopods, potentially indicating behavioural differences between the clades. Hadrosaurs show more cursorial adaptations than other quadrupedal ornithischians, possibly reflecting different behavioural strategies related to home range size, migration or predator escape.

## Supporting Information

Supporting Information S1 **Raw data used in statistical analyses; stratigraphic data used to calculate branch lengths; the results of K-means cluster analysis.**
(DOC)
