# Supplementary material for: Limb-Bone Scaling Indicates Diverse Stance and Gait in Quadrupedal Ornithischian Dinosaurs
Source: PLoS One. 2012 May 22;7(5):e36904. doi: 10.1371/journal.pone.0036904 (PMC3358279; doi:10.1371/journal.pone.0036904)
Supplement: Supporting Information S1 — Raw data used in statistical analyses; stratigraphic data used to calculate branch lengths; the results of K-means cluster analysis. (DOC) [file pone.0036904.s001.doc]

**Supporting Information S1—Limb Bone Scaling Indicates Diverse Stance and Gait in Quadrupedal Ornithischian Dinosaurs**

## Authors and Affiliations

**Susannah C. R. Maidment**^*^, Department of Palaeontology, Natural History Museum, Cromwell Road London, SW7 5BD, United Kingdom.

**Deborah H. Linton**, Department of Earth Sciences, University College London, Gower Street, London, WC1E 6BT, United Kingdom and Department of Palaeontology, Natural History Museum, Cromwell Road London, SW7 5BD, United Kingdom.

**Paul Upchurch**, Department of Earth Sciences, University College London, Gower Street, London, WC1E 6BT, United Kingdom.

**Paul M. Barrett**, Department of Palaeontology, Natural History Museum, Cromwell Road London, SW7 5BD, United Kingdom.

*Corresponding author: [s.maidment@nhm.ac.uk](mailto:s.maidment@nhm.ac.uk)

**1. Institutional Abbreviations**

**AMNH**, American Museum of Natural History, New York, USA; **BINS**, Royal Belgian Institute of Natural Sciences, Brussels, Belgium; **BYU**, Brigham Young University Museum, Provo, Utah, USA; **CM**, Carnegie Museum, Pittsburgh, USA; **CMN**, Canadian Museum of Nature, Ottawa, Canada; **Fukui**, Fukui Prefectural Dinosaur Museum, Fukui, Japan; **IVPP**, Institute of Vertebrate Paleontology and Paleoanthropology, Beijing, China; **MANCH**, Manchester Museum, UK; **MB**, Museum für Naturkunde, Berlin, Germany; **NHMUK**, Natural History Museum, London, UK; **NMST**, National Museum of Science, Tokyo, Japan; **ROM**, Royal Ontario Museum, Toronto, Canada; **SMA**, Sauriermuseum, Aathal, Switzerland; **TMP**, Royal Tyrrell Museum of Paleontology, Drumheller, Canada; **USNM**, National Museum of Natural History, Smithsonian Institution, Washington D.C., USA; **YPM**, Peabody Museum, Yale University, New Haven, USA; **ZDM**, Zigong Dinosaur Museum, Zigong, China.

Specimens housed at Fukui were all casts of specimens from other institutions and did not have specimen numbers. YPM specimens followed by the number PUXXX were Princeton University specimens housed at the Peabody Museum.

**2. Raw Data**

**Table S1. Specimens, phylogenetic groupings and measurements used in regressions of femoral proportions**

| **Taxon** | **Grouping** | **Specimen** | **Length (mm)** | **AP width (mm)** | **ML width (mm)** |
| --- | --- | --- | --- | --- | --- |
| Ankylosauria indet. | Ankylosauria | NHMUK R1609 | 310 | 44 | 55 |
| *Ankylosaurus magniventris* | Ankylosauria | AMNH 5214 | 650 | 89 | 130 |
| *Dyoplosaurus acutosquameus* | Ankylosauria | ROM 784 | 515 | 124 | 100 |
| *Hoplitosaurus marshi* | Ankylosauria | USNM 4752 | 479 | 116 | 96 |
| *Panoplosaurus mirus* | Ankylosauria | AMNH 3072 | 410 | 89 | 67 |
| *Sauropelta edwardsi* | Ankylosauria | AMNH 3032 | 723 | 81 | 125 |
| *Struthiosaurus austriacus* | Ankylosauria | CM 971 | 260 | 28 | 39 |
| *Abrictosaurus consors* | Bipedal ornithischian | NHMUK RUB 54 | 78 | 8 | 6 |
| *Agilisaurus louderbacki* | Bipedal ornithischian | Fukui *Agilisaurus* | 114 | 15 | 15 |
| *Fulgotherium australe* | Bipedal ornithischian | NHMUK R12209 | 149 | 19 | 11 |
| *Heterodontosaurus tucki* | Bipedal ornithischian | USNM 299765 | 112 | 13 | 10 |
| *Hypsilophodon foxii* | Bipedal ornithischian | NHMUK R192 | 178 | 23 | 22 |
| *Hypsilophodon foxii* | Bipedal ornithischian | NHMUK R196 | 147 | 18 | 14 |
| *Hypsilophodon foxii* | Bipedal ornithischian | NHMUK R5830 | 99 | 11 | 11 |
| *Jeholosaurus shangyuanensis* | Bipedal ornithischian | IVPP V12529 | 91 | 10 | 9 |
| *Jeholosaurus shangyuanensis* | Bipedal ornithischian | IVPP V15939 | 133 | 17 | 14 |
| *Lesothosaurus diagnosticus* | Bipedal ornithischian | NHMUK RUB 17 | 102 | 12 | 12 |
| *Othneilosaurus consors* | Bipedal ornithischian | USNM 8397 | 162 | 18 | 18 |
| *Othneilosaurus consors* | Bipedal ornithischian | YPM 4754 | 142 | 22 | 15 |
| *Parksosaurus warreni* | Bipedal ornithischian | ROM 804 | 263 | 37 | 25 |
| *Thescelosaurus neglectus* | Bipedal ornithischian | AMNH 973 | 330 | 43 | 48 |
| *Thescelosaurus neglectus* | Bipedal ornithischian | NHMUK unregistered | 485 | 55 | 74 |
| *Thescelosaurus neglectus* | Bipedal ornithischian | NMST *Thescelosaurus* | 343 | 46 | 51 |
| *Centrosaurus apertus* | Ceratopsidae | ROM 1426 | 708 | 121 | 124 |
| Ceratopsidae indet. | Ceratopsidae | NHMUK unregistered | 840 | 90 | 150 |
| Ceratopsidae indet. | Ceratopsidae | USNM 14765 | 585 | 73 | 73 |
| *Chasmosaurus belli* | Ceratopsidae | NHMUK R4948 | 780 | 80 | 123 |
| *Chasmosaurus* sp. | Ceratopsidae | ROM 839 | 730 | 190 | 103 |
| *Chasmosaurus belli* | Ceratopsidae | ROM 843 | 840 | 100 | 135 |
| *Pachyrhinosaurus* sp. | Ceratopsidae | TMP 2002.76.01 | 905 | 120 | 152 |
| *Pachyrhinosaurus canadensis* | Ceratopsidae | TMP 87.55.30 | 430 | 56 | 40 |
| *Pachyrhinosaurus canadensis* | Ceratopsidae | TMP 87.55.307 | 420 | 27 | 58 |
| *Pachyrhinosaurus canadensis* | Ceratopsidae | TMP 88.55.17 | 360 | 54 | 25 |
| *Pachyrhinosaurus canadensis* | Ceratopsidae | TMP 89.55.429 | 625 | 43 | 110 |
| *Triceratops horridus* | Ceratopsidae | AMNH 5033 | 1040 | 140 | 110 |
| *Triceratops horridus* | Ceratopsidae | USNM *Triceratops* | 870 | 81 | 119 |
| *Triceratops horridus* | Ceratopsidae | YPM 1821 | 935 | 95 | 165 |
| *Corythosaurus casuarius* | Hadrosauridae | USNM 11893 | 640 | 55 | 93 |
| *Corythosaurus casuarius* | Hadrosauridae | USNM 15578 | 585 | 48 | 91 |
| *Corythosaurus casuarius* | Hadrosauridae | USNM 358591 | 500 | 39 | 66 |
| *Corythosaurus casuarius* | Hadrosauridae | USNM 358593 | 533 | 57 | 76 |
| *Corythosaurus casuarius* | Hadrosauridae | USNM 358594 | 588 | 118 | 74 |
| *Edmontosaurus* sp. | Hadrosauridae | CM 11745 | 900 | 110 | 121 |
| *Edmontosaurus* sp. | Hadrosauridae | NHMUK unregistered | 1050 | 165 | 111 |
| *Edmontosaurus annectens* | Hadrosauridae | ROM 801 | 1250 | 295 | 120 |
| *Edmontosaurus regalis* | Hadrosauridae | CMN 2289 | 1260 | 171 | 192 |
| *Gryposaurus notabilis* | Hadrosauridae | AMNH 5350 | 1100 | 127 | 193 |
| *Gryposaurus notabilis* | Hadrosauridae | AMNH 5465 | 1180 | 160 | 125 |
| Hadrosauridae indet. | Hadrosauridae | ROM 656 | 1045 | 164 | 150 |
| Hadrosauridae indet. | Hadrosauridae | TMP 87.78.5 | 550 | 51 | 69 |
| *Hadrosaurus foulkii* | Hadrosauridae | TMP 84.181.1 | 960 | 108 | 118 |
| *Hypacrosaurus altispinus* | Hadrosauridae | AMNH 5217 | 940 | 99 | 108 |
| *Hypacrosaurus altispinus* | Hadrosauridae | AMNH 5357 | 795 | 91 | 98 |
| *Hypacrosaurus altispinus* | Hadrosauridae | TMP 2007.10.2 | 505 | 71 | 46 |
| *Lambeosaurus lambei* | Hadrosauridae | ROM 1218 | 1080 | 120 | 131 |
| *Maiasaura peeblesorum* | Hadrosauridae | ROM 44770 | 990 | 155 | 116 |
| *Nipponsaurus sachalinensis* | Hadrosauridae | NMST *Nipponosaurus* | 538 | 53 | 55 |
| *Parasaurolophus walkeri* | Hadrosauridae | ROM 876 | 1030 | 155 | 90 |
| *Huayangosaurus taibaii* | Stegosauria | ZDM T7001 | 510 | 62 | 85 |
| *Kentrosaurus aethiopicus* | Stegosauria | MB 4800 | 625 | 69 | 101 |
| *Kentrosaurus aethiopicus* | Stegosauria | MB R.3576 | 625 | 65 | 97 |
| *Kentrosaurus aethiopicus* | Stegosauria | MB R.3579 | 585 | 62 | 76 |
| *Kentrosaurus aethiopicus* | Stegosauria | MB R.3582 | 650 | 68 | 91 |
| *Kentrosaurus aethiopicus* | Stegosauria | MB R.3594 | 660 | 70 | 90 |
| *Loricatosaurus priscus* | Stegosauria | NHMUK R3167 | 890 | 83 | 111 |
| Stegosauria indet. | Stegosauria | NHMUK R1992 | 1005 | 85 | 135 |
| *Stegosaurus armatus* | Stegosauria | AMNH 650 | 1050 | 70 | 170 |
| *Stegosaurus armatus* | Stegosauria | CM 21709 | 960 | 71 | 129 |
| *Stegosaurus armatus* | Stegosauria | CM 21758 | 850 | 48 | 114 |
| *Stegosaurus armatus* | Stegosauria | CM 21770 | 1110 | 100 | 150 |
| *Stegosaurus armatus* | Stegosauria | CM 33919 | 1050 | 78 | 142 |
| *Stegosaurus armatus* | Stegosauria | CM 36696 | 1015 | 90 | 135 |
| *Stegosaurus armatus* | Stegosauria | CM 557 | 840 | 71 | 105 |
| *Stegosaurus armatus* | Stegosauria | YPM 1387 | 950 | 52 | 115 |
| *Stegosaurus armatus* | Stegosauria | YPM 1856 | 840 | 67 | 109 |
| *Stegosaurus armatus* | Stegosauria | YPM 4634 | 500 | 44 | 64 |
| *Tuojiangosaurus multispinus* | Stegosauria | Fukui *Tuojiangosaurus* | 810 | 56 | 138 |
| *Bactrosaurus johnsoni* | Ornithopoda | Fukui *Bactrosaurus* | 540 | 75 | 82 |
| *Camptosaurus dispar* | Ornithopoda | AMNH 959 | 260 | 38 | 44 |
| *Camptosaurus dispar* | Ornithopoda | CM 15780 | 345 | 57 | 53 |
| *Camptosaurus dispar* | Ornithopoda | CM 21722 | 310 | 41 | 44 |
| *Camptosaurus dispar* | Ornithopoda | CM 21723 | 320 | 56 | 53 |
| *Camptosaurus dispar* | Ornithopoda | USNM 5818 | 578 | 78 | 111 |
| *Camptosaurus dispar* | Ornithopoda | YPM 1877 | 550 | 114 | 104 |
| *Mantellisaurus atherfieldensis* | Ornithopoda | BINS 1551 | 720 | 183 | 108 |
| *Dryosaurus altus* | Ornithopoda | AMNH 834 | 215 | 24 | 22 |
| *Dryosaurus altus* | Ornithopoda | USNM 5830 | 345 | 39 | 38 |
| *Dryosaurus altus* | Ornithopoda | YPM 1876 | 370 | 47 | 40 |
| *Gilmoreosaurus mongoliensis* | Ornithopoda | AMNH 6551 | 680 | 69 | 95 |
| *Iguanodon bernissartensis* | Ornithopoda | BINS 1534 | 1000 | 130 | 155 |
| *Iguanodon bernissartensis* | Ornithopoda | BINS 1713 | 1048 | 181 | 149 |
| *Iguanodon bernissartensis* | Ornithopoda | BINS 1731 | 970 | 135 | 155 |
| *Muttaburrasaurus langdoni* | Ornithopoda | Fukui *Muttaburrasaurus* | 980 | 126 | 137 |
| Ornithopoda indet. | Ornithopoda | ROM 45959 | 88 | 7 | 9 |
| Ornithopoda indet. | Ornithopoda | ROM 45962 | 100 | 14 | 12 |
| Ornithopoda indet. | Ornithopoda | ROM 45969 | 175 | 21 | 18 |
| Ornithopoda indet. | Ornithopoda | ROM 45970 | 128 | 16 | 14 |
| Ornithopoda indet. | Ornithopoda | TMP 19.11.32 | 170 | 20 | 19 |
| *Ouranosaurus nigeriensis* | Ornithopoda | Fukui *Ouranosaurus* | 880 | 86 | 111 |
| *Tenontosaurus tilletti* | Ornithopoda | AMNH 3014 | 315 | 40 | 45 |
| *Tenontosaurus tilletti* | Ornithopoda | AMNH 3022 | 210 | 23 | 26 |
| *Tenontosaurus tilletti* | Ornithopoda | AMNH 3031 | 365 | 46 | 54 |
| *Tenontosaurus tilletti* | Ornithopoda | AMNH 3040 | 460 | 103 | 71 |
| *Montanoceratops cerorhynchus* | Marginocephalia | AMNH 5464 | 338 | 58 | 46 |
| *Pachycephalosaurus wyomingensis* | Marginocephalia | Fukui *Pachycephalosaurus* | 468 | 47 | 60 |
| *Psittacosaurus mongoliensis* | Marginocephalia | AMNH 6541 | 175 | 23 | 20 |
| *Scelidosaurus harrisoni* | Thyreophora | NHMUK R1111 | 410 | 57 | 53 |

**Table 2. Specimens, phylogenetic groupings and measurements used in regressions of humeral proportions.**

| **Taxon** | **Grouping** | **Specimen** | **Length (mm)** | **AP width (mm)** | **ML width (mm)** | **DPC width (mm)** |
| --- | --- | --- | --- | --- | --- | --- |
| *Ankylosaurus magniventris* | Ankylosauria | AMNH 5214 | 563 | 74 | 97 | 218 |
| *Edmontonia longiceps* | Ankylosauria | CMN 8531 | 485 | 49 | 98 | 200 |
| *Edmontonia longiceps* | Ankylosauria | ROM 1215 | 410 | 49 | 79 | 139 |
| *Euoplocephalus tutus* | Ankylosauria | AMNH 5337 | 413 | 71 | 76 | 173 |
| *Euoplocephalus tutus* | Ankylosauria | AMNH 5403 | 495 | 54 | 82 | 135 |
| *Euoplocephalus tutus* | Ankylosauria | AMNH 5404 | 410 | 61 | 81 | 155 |
| *Euoplocephalus tutus* | Ankylosauria | AMNH 5405 | 445 | 74 | 76 | 135 |
| *Euoplocephalus tutus* | Ankylosauria | ROM 47655 | 460 | 69 | 80 | 190 |
| *Gastonia burgei* | Ankylosauria | BYU 15502 | 300 |  | 46 | 118 |
| *Panoplosaurus mirus* | Ankylosauria | CMN 2759 | 440 | 53 | 72 | 240 |
| *Sauropelta edwardsi* | Ankylosauria | AMNH 3016 | 520 | 75 | 74 | 150 |
| *Sauropelta edwardsi* | Ankylosauria | AMNH 3032 | 530 | 72 | 81 | 250 |
| *Sauropelta edwardsi* | Ankylosauria | AMNH 5853 | 525 | 48 | 64 | 115 |
| *Agilisaurus louderbacki* | Bipedal ornithischian | Fukui *Agilisaurus* | 116 | 11 | 12 | 22 |
| *Heterodontosaurus tucki* | Bipedal ornithischian | USNM 299765 | 80 | 7 | 6 | 12 |
| *Hypsilophodon foxii* | Bipedal ornithischian | NHMUK R194 | 106 | 10 | 11 | 18 |
| *Hypsilophodon foxii* | Bipedal ornithischian | NHMUK R196 | 104 | 10 | 11 | 18 |
| *Lesothosaurus diagnosticus* | Bipedal ornithischian | NHMUK RUB 17 | 66 | 6 | 7 | 12 |
| *Parksosaurus warreni* | Bipedal ornithischian | ROM 804 | 215 | 14 | 23 | 53 |
| *Thescelosaurus neglectus* | Bipedal ornithischian | AMNH 5031 | 210 | 21 | 24 | 32 |
| *Thescelosaurus neglectus* | Bipedal ornithischian | AMNH 5034 | 320 | 31 | 38 | 59 |
| *Thescelosaurus neglectus* | Bipedal ornithischian | AMNH 5891 | 320 | 28 | 36 | 55 |
| *Thescelosaurus neglectus* | Bipedal ornithischian | AMNH 5893 | 690 | 83 | 102 | 220 |
| *Thescelosaurus neglectus* | Bipedal ornithischian | NMST *Thescelosaurus* | 225 | 21 | 28 | 36 |
| *Centrosaurus apertus* | Ceratopsidae | ROM 767 | 538 | 49 | 61 | 152 |
| Ceratopsidae indet. | Ceratopsidae | AMNH 3998 | 565 | 48 | 88 | 160 |
| Ceratopsidae indet. | Ceratopsidae | CMN 9812 | 350 | 49 | 64 | 123 |
| Ceratopsidae indet. | Ceratopsidae | CMN 8547 | 590 | 78 | 62 | 170 |
| Ceratopsidae indet. | Ceratopsidae | TMP 89.97.1 | 520 | 47 | 79 | 165 |
| Ceratopsidae indet. | Ceratopsidae | TMP BB138-21 | 550 | 69 | 80 | 129 |
| *Chasmosaurus belli* | Ceratopsidae | CMN 2245 | 500 | 63 | 74 | 150 |
| *Chasmosaurus belli* | Ceratopsidae | NHMUK R4948 | 590 | 76 | 79 | 170 |
| *Chasmosaurus* sp. | Ceratopsidae | ROM 839 | 580 | 70 | 82 | 233 |
| *Pachyrhinosaurus* sp. | Ceratopsidae | TMP 2002.76.01 | 690 | 70 | 88 | 158 |
| *Pachyrhinosaurus canadensis* | Ceratopsidae | TMP 86.55.186 | 305 | 30 | 38 | 77 |
| *Pachyrhinosaurus canadensis* | Ceratopsidae | TMP 87.55.265 | 310 | 24 | 36 | 71 |
| *Pachyrhinosaurus canadensis* | Ceratopsidae | TMP 89.55.250 | 205 | 13 | 25 | 58 |
| *Pachyrhinosaurus canadensis* | Ceratopsidae | TMP 89.55.426 | 415 | 21 | 56 | 113 |
| *Pachyrhinosaurus canadensis* | Ceratopsidae | TMP 89.55.43 | 305 | 32 | 28 | 66 |
| *Pachyrhinosaurus canadensis* | Ceratopsidae | TMP 89.55.510 | 305 | 35 | 38 | 73 |
| *Pachyrhinosaurus canadensis* | Ceratopsidae | TMP 89.55.695 | 300 | 17 | 37 | 60 |
| *Styracosaurus albertensis* | Ceratopsidae | CMN 344 | 600 | 80 | 85 | 180 |
| *Triceratops horridus* | Ceratopsidae | AMNH 5039 | 825 | 75 | 155 | 320 |
| *Triceratops horridus* | Ceratopsidae | AMNH 971 | 830 | 78 | 190 | 300 |
| *Triceratops horridus* | Ceratopsidae | CM 1618 | 680 | 88 | 124 | 230 |
| *Triceratops horridus* | Ceratopsidae | USNM 4276 | 847 | 103 | 145 | 280 |
| *Triceratops horridus* | Ceratopsidae | USNM 8018 | 855 | 90 | 180 | 320 |
| *Brachylophosaurus canadensis* | Hadrosauridae | CMN 8893 | 588 | 56 | 72 | 122 |
| *Corythosaurus casuarius* | Hadrosauridae | USNM 358594 | 284 | 23.3 | 41.8 | 71.7 |
| *Edmontosaurus regalis* | Hadrosauridae | CMN 2289 | 690 | 67 | 89 | 200 |
| *Edmontosaurus annectens* | Hadrosauridae | ROM 801 | 675 | 81 | 59 | 185 |
| *Gryposaurus notabilis* | Hadrosauridae | AMNH 5465 | 740 | 53 | 93 | 150 |
| *Gryposaurus notabilis* | Hadrosauridae | ROM 764 | 580 | 90 | 63 | 149 |
| *Gryposaurus notabilis* | Hadrosauridae | USNM 358554 | 295 | 27 | 41 | 69 |
| Hadrosaurdae indet. | Hadrosauridae | CMN 9718 | 640 | 36 | 93 | 150 |
| Hadrosauridae indet. | Hadrosauridae | AMNH 5224 | 335 | 37 | 50 | 175 |
| Hadrosauridae indet. | Hadrosauridae | CM 1066 | 635 | 56 | 70 | 136 |
| Hadrosauridae indet. | Hadrosauridae | CMN 40603 | 560 | 72 | 78 | 131 |
| Hadrosauridae indet. | Hadrosauridae | CMN 419 | 690 | 73 | 94 | 155 |
| Hadrosauridae indet. | Hadrosauridae | CMN 9584 | 415 | 38 | 52 | 94 |
| Hadrosauridae indet. | Hadrosauridae | CMN 9795 | 420 | 33 | 55 | 92 |
| Hadrosauridae indet. | Hadrosauridae | CMN 9800 | 515 | 36 | 75 | 123 |
| Hadrosauridae indet. | Hadrosauridae | CMN WL125.1 | 555 | 45 | 74 | 142 |
| Hadrosauridae indet. | Hadrosauridae | CMN WL126.161 | 525 | 36 | 73 | 130 |
| Hadrosauridae indet. | Hadrosauridae | ROM 3508 | 210 | 27 | 30 | 58 |
| Hadrosauridae indet. | Hadrosauridae | ROM 49695 | 530 | 60 | 74 | 151 |
| Hadrosauridae indet. | Hadrosauridae | TMP 80.29.101 | 480 | 69 | 75 | 134 |
| Hadrosauridae indet. | Hadrosauridae | TMP 92.30.47 | 460 | 48 | 58 | 104 |
| Hadrosauridae indet. | Hadrosauridae | TMP 93.36.331 | 365 | 34 | 53 | 91 |
| *Hadrosaurus foulkii* | Hadrosauridae | TMP 84.181.1 | 570 | 72 | 76 | 130 |
| *Hypacrosaurus altispinus* | Hadrosauridae | AMNH 5357 | 413 | 42 | 57 | 113 |
| *Hypacrosaurus altispinus* | Hadrosauridae | USNM 11950 | 585 | 41.6 | 92.5 | 169 |
| *Maiasaura peeblesorum* | Hadrosauridae | ROM 44771 | 230 | 18 | 25 | 43 |
| *Nipponsaurus sachalinensis* | Hadrosauridae | NMST *Nipponosaurus* | 265 | 26 | 46 | 67 |
| *Parasaurolphus walkeri* | Hadrosauridae | ROM 768 | 505 | 54 | 88 | 170 |
| *Parasaurolphus walkeri* | Hadrosauridae | TMP 92.53.1 | 545 | 69 | 73 | 144 |
| *Prosaurolophus maximus* | Hadrosauridae | ROM 787 | 520 | 54 | 65 | 135 |
| *Saurolophus osborni* | Hadrosauridae | AMNH 5225 | 680 | 84 | 109 | 210 |
| *Saurolophus osborni* | Hadrosauridae | AMNH 5271 | 465 | 38 | 177 | 126 |
| *Dacentrurus armatus* | Stegosauria | NHMUK 46013 | 850 | 99 | 134 | 300 |
| *Kentrosaurus aethiopicus* | Stegosauria | MB R.4804 | 350 | 53 | 66 | 128 |
| *Kentrosaurus aethiopicus* | Stegosauria | MB R.4805 | 385 | 61 | 74 |  |
| *Loricatosaurus priscus* | Stegosauria | NHMUK R3167 | 525 | 68 | 91 | 225 |
| Stegosauria indet. | Stegosauria | NHMUK R5902 | 720 | 97 | 135 | 285 |
| *Stegosaurus armatus* | Stegosauria | AMNH 464 | 515 | 81 | 104 | 220 |
| *Stegosaurus armatus* | Stegosauria | CM 21737 | 530 | 72 | 101 | 230 |
| *Stegosaurus armatus* | Stegosauria | USNM 4936 | 540 | 80 | 103 | 175 |
| *Stegosaurus armatus* | Stegosauria | USNM 4937 | 590 | 121 | 87 | 290 |
| *Stegosaurus armatus* | Stegosauria | USNM 6539 | 600 | 82 | 107 | 200 |
| *Stegosaurus armatus* | Stegosauria | YPM 1284/1304 | 470 | 75 | 104 | 190 |
| *Stegosaurus armatus* | Stegosauria | YPM 1387 | 540 | 79 | 104 | 320 |
| *Stegosaurus armatus* | Stegosauria | YPM 1387/1512 | 550 | 87 | 114 | 310 |
| *Stegosaurus armatus* | Stegosauria | YPM 1394 | 435 | 61 | 93 | 155 |
| *Stegosaurus armatus* | Stegosauria | YPM 1856 | 430 | 71 | 94 | 220 |
| *Stegosaurus armatus* | Stegosauria | YPM 4634 | 270 | 39 | 50 | 140 |
| *Tuojiangosaurus multispinus* | Stegosauria | Fukui *Tuojiangosaurus* | 550 | 66 | 81 | 225 |
| *Bactrosaurus johnsoni* | Ornithopoda | Fukui *Bactrosaurus* | 215 | 29 | 32 | 45 |
| *Camptosaurus dispar* | Ornithopoda | AMNH 596 | 430 | 39 | 56 | 79 |
| *Camptosaurus dispar* | Ornithopoda | AMNH 6111 | 260 | 31 | 37 | 54 |
| *Camptosaurus dispar* | Ornithopoda | AMNH 646 | 245 | 24 | 31 | 49 |
| *Camptosaurus dispar* | Ornithopoda | USNM 4282 | 360 | 37 | 45 | 66 |
| *Camptosaurus dispar* | Ornithopoda | USNM 5473 | 368 | 43 | 50 | 72 |
| *Camptosaurus dispar* | Ornithopoda | YPM 6794 | 250 | 29 | 37 | 49 |
| *Mantellisaurus atherfieldensis* | Ornithopoda | BINS 1551 | 433 | 47 | 46 | 99 |
| *Dryosaurus altus* | Ornithopoda | YPM 1876 | 190 | 17 | 20 | 32 |
| *Gasparinisaura cincolsaltensis* | Ornithopoda | NMST 20392 | 52 | 5 | 4 | 6 |
| *Iguanodon bernissartensis* | Ornithopoda | BINS 1534 | 790 | 84 | 109 | 175 |
| *Iguanodon bernissartensis* | Ornithopoda | BINS 1536 | 770 | 82 | 122 | 160 |
| *Iguanodon bernissartensis* | Ornithopoda | BINS 1562 | 855 | 63 | 160 | 220 |
| *Iguanodon bernissartensis* | Ornithopoda | BINS 1657 | 805 | 79 | 139 | 220 |
| *Iguanodon bernissartensis* | Ornithopoda | BINS 1715 | 920 | 78 | 126 | 175 |
| *Iguanodon bernissartensis* | Ornithopoda | BINS 1729 | 580 | 75 | 93 | 124 |
| *Muttaburrasaurus langdoni* | Ornithopoda | Fukui *Muttaburrasaurus* | 650 | 79 | 90 | 125 |
| Ornithopoda indet. | Ornithopoda | NMST 684 | 225 | 13 | 23 | 39 |
| *Ouranosaurus nigeriensis* | Ornithopoda | Fukui *Ouranosaurus* | 485 | 50 | 58 | 85 |
| *Tenontosaurus tilletti* | Ornithopoda | AMNH 3014 | 245 | 25 | 32 | 66 |
| *Tenontosaurus tilletti* | Ornithopoda | AMNH 3022 | 158 | 18 | 19 | 39 |
| *Tenontosaurus tilletti* | Ornithopoda | AMNH 3043 | 260 | 26 | 29 | 67 |
| *Tenontosaurus tilletti* | Ornithopoda | YPM PU 16514 | 195 | 22 | 21 | 27 |
| *Tenontosaurus tilletti* | Ornithopoda | USNM 466042 | 285 | 28 | 40 | 79 |
| *Tenontosaurus tilletti* | Ornithopoda | YPM 5456 | 438 | 53 | 57 | 120 |
| *Tenontosaurus tilletti* | Ornithopoda | YPM BB1 | 270 | 28 | 35 | 74 |
| *Pachycephalosaurus wyomingensis* | Marginocephalia | Fukui *Pachcephalosaurus* | 213 | 21 | 24 | 28 |
| *Protoceratops andrewsi* | Marginocephalia | AMNH 6424 | 210 | 20 | 20 | 51 |
| *Psittacosaurus mongoliensis* | Marginocephalia | AMNH 6544 | 108 | 10 | 11 | 26 |
| *Psittacosaurus neimongoliensis* | Marginocephalia | IVPP 120888 | 106 | 10 | 11 | 27 |
| *Leptoceratops gracilis* | Marginocephalia | AMNH 5205 | 298 | 33 | 35 | 72 |

**Table S3. Specimens, phylogenetic groupings and measurements used in regressions of ulna proportions.**

| **Taxon** | **Grouping** | **Specimen number** | **Length (mm)** | **AP width (mm)** | **ML width (mm)** |
| --- | --- | --- | --- | --- | --- |
| *Edmontonia longiceps* | Ankylosauria | ROM 1215 | 350 | 28 | 115 |
| *Edmontonia longiceps* | Ankylosauria | TMP 98.98.1 | 415 | 113 | 205 |
| *Euoplocephalus tutus* | Ankylosauria | AMNH 5405 | 395 | 125 | 160 |
| *Euoplocephalus tutus* | Ankylosauria | AMNH 5406 | 320 | 70 | 145 |
| *Sauropelta edwardsi* | Ankylosauria | AMNH 3032 | 485 | 148 | 188 |
| *Sauropelta edwardsi* | Ankylosauria | AMNH 3035 | 490 | 170 | 185 |
| *Agilisaurus louderbacki* | Bipedal ornithischian | Fukui *Agilisaurus* | 86 | 15 | 18 |
| *Parksosaurus warreni* | Bipedal ornithischian | ROM 804 | 142 | 19 | 45 |
| *Thescelosaurus neglectus* | Bipedal ornithischian | AMNH 5034 | 210 | 50 | 60 |
| *Thescelosaurus neglectus* | Bipedal ornithischian | AMNH 5043 | 620 | 180 | 210 |
| *Thescelosaurus neglectus* | Bipedal ornithischian | NMST *Thescelosaurus* | 163 | 28 | 34 |
| *Centrosaurus apertus* | Ceratopsidae | ROM 1426 | 410 | 90 | 129 |
| *Centrosaurus apertus* | Ceratopsidae | ROM 767 | 415 | 80 | 170 |
| *Centrosaurus apertus* | Ceratopsidae | TMP 87.36.160 | 350 | 90 | 103 |
| *Centrosaurus apertus* | Ceratopsidae | USNM 12745 | 506 | 95 | 161 |
| Ceratopsidae indet. | Ceratopsidae | CMN 8547 | 450 | 28 | 148 |
| *Chasmosaurus belli* | Ceratopsidae | CMN 2245 | 440 | 74 | 159 |
| *Chasmosaurus* sp. | Ceratopsidae | ROM 839 | 435 | 113 | 137 |
| *Chasmosaurus belli* | Ceratopsidae | ROM 843 | 493 | 83 | 185 |
| *Pachyrhinosaurus canadensis* | Ceratopsidae | TMP 87.55.176 | 210 | 33 | 66 |
| *Pachyrhinosaurus canadensis* | Ceratopsidae | TMP 87.55.200 | 390 | 60 | 145 |
| *Pachyrhinosaurus canadensis* | Ceratopsidae | TMP 89.55.227 | 400 | 49 | 153 |
| *Triceratops horridus* | Ceratopsidae | AMNH 970 | 645 | 140 | 260 |
| *Triceratops horridus* | Ceratopsidae | AMNH 971 | 645 | 185 | 290 |
| *Triceratops horridus* | Ceratopsidae | CM 1618 | 605 | 82 | 240 |
| *Triceratops horridus* | Ceratopsidae | CM 998 | 635 | 185 | 245 |
| *Triceratops horridus* | Ceratopsidae | USNM 4848 | 683 | 200 | 256 |
| *Brachylophosaurus canadensis* | Hadrosauridae | CMN 8893 | 713 | 91 | 118 |
| *Corythosaurus casuarius* | Hadrosauridae | USNM 11893 | 428 | 60 | 83 |
| *Corythosaurus casuarius* | Hadrosauridae | USNM 358588 | 340 | 53.3 | 48.7 |
| *Edmontosaurus* sp. | Hadrosauridae | CM 30745 | 465 | 50 | 81 |
| *Edmontosaurus regalis* | Hadrosauridae | CMN 2289 | 768 | 100 | 142 |
| *Edmontosaurus* sp. | Hadrosauridae | CMN 419 | 710 | 66 | 135 |
| *Edmontosaurus annectens* | Hadrosauridae | ROM 801 | 750 | 108 | 151 |
| *Edmontosaurus* sp. | Hadrosauridae | USNM 3814 | 650 | 137 | 135 |
| *Edmontosaurus* sp. | Hadrosauridae | USNM 4278 | 665 | 121 | 135 |
| *Gryposaurus notabilis* | Hadrosauridae | ROM 764 | 610 | 100 | 103 |
| *Gryposaurus notabilis* | Hadrosauridae | USNM 214588 | 340 | 43 | 64 |
| *Gryposaurus notabilis* | Hadrosauridae | USNM 358554 | 360 | 33 | 57 |
| *Gryposaurus notabilis* | Hadrosauridae | YPM PU16969 | 635 | 83 | 100 |
| Hadrosauridae indet. | Hadrosauridae | AMNH 5212 | 750 | 100 | 145 |
| Hadrosauridae indet. | Hadrosauridae | TMP 05.09.84 | 355 | 46 | 71 |
| Hadrosauridae indet. | Hadrosauridae | TMP 67.10.79 | 455 | 66 | 77 |
| Hadrosauridae indet. | Hadrosauridae | TMP 82.16.264 | 215 | 90 | 106 |
| Hadrosauridae indet. | Hadrosauridae | TMP 90.36.421 | 400 | 67 | 73 |
| *Hadrosaurus foulki* | Hadrosauridae | AMNH 5472 | 490 | 49 | 72 |
| *Hadrosaurus foulki* | Hadrosauridae | CMN 40603 | 590 | 95 | 120 |
| *Hadrosaurus foulki* | Hadrosauridae | TMP 84.181.1 | 590 | 92 | 120 |
| *Hypacrosaurus altispinus* | Hadrosauridae | AMNH 5272 | 745 | 120 | 139 |
| *Hypacrosaurus altispinus* | Hadrosauridae | AMNH 5357 | 513 | 33 | 95 |
| *Hypacrosaurus altispinus* | Hadrosauridae | USNM 7948 | 673 | 90 | 114 |
| *Hypacrosaurus altispinus* | Hadrosauridae | USNM 7948.2 | 674 | 90 | 121 |
| *Maiasaura peeblesorum* | Hadrosauridae | YPM PU22400 | 84 | 11 | 14 |
| *Nipponsaurus sachalinensis* | Hadrosauridae | NMST *Nipponosaurus* | 263 | 41 | 52 |
| *Parasaurolophus walkeri* | Hadrosauridae | ROM 768 | 560 | 97 | 124 |
| *Prosaurolophus maximus* | Hadrosauridae | ROM 1423 | 630 | 98 | 93 |
| *Prosaurolophus maximus* | Hadrosauridae | ROM 787 | 550 | 89 | 120 |
| *Saurolophus osborni* | Hadrosauridae | AMNH 5271 | 468 | 86 | 89 |
| *Stegosaurus armatus* | Stegosauria | USNM 4937 | 586 | 107 | 206 |
| *Stegosaurus armatus* | Stegosauria | USNM 7401 | 371 | 67.9 | 146 |
| *Stegosaurus armatus* | Stegosauria | USNM 7754 | 605 | 110 | 200 |
| *Stegosaurus armatus* | Stegosauria | YPM 1365/1855/6 | 475 | 126 | 175 |
| *Stegosaurus armatus* | Stegosauria | YPM 1855/6 | 390 | 119 | 160 |
| *Stegosaurus armatus* | Stegosauria | YPM 4836 | 530 | 138 | 145 |
| *Tuojiangosaurus multispinus* | Stegosauria | Fukui *Tuojiangosaurus* | 505 | 175 | 188 |
| *Bactrosaurus osborni* | Ornithopoda | Fukui *Bactrosaurus* | 235 | 35 | 47 |
| *Camptosaurus dispar* | Ornithopoda | AMNH 586 | 295 | 60 | 96 |
| *Camptosaurus dispar* | Ornithopoda | USNM 4282 | 265 | 48 | 74 |
| *Camptosaurus dispar* | Ornithopoda | USNM 4697 | 265 | 56 | 74 |
| *Camptosaurus dispar* | Ornithopoda | USNM 5473 | 278 | 73 | 79 |
| *Camptosaurus dispar* | Ornithopoda | USNM 5825 | 260 | 60 | 84 |
| *Camptosaurus dispar* | Ornithopoda | YPM 1878 | 260 | 58 | 80 |
| *Camptosaurus dispar* | Ornithopoda | YPM 6794 | 195 | 46 | 57 |
| *Camptosaurus dispar* | Ornithopoda | YPM 8616 | 230 | 45 | 62 |
| *Mantellisaurus atherfieldensis* | Ornithopoda | BINS 1551 | 395 | 83 | 63 |
| *Dryosaurus alus* | Ornithopoda | YPM 1876 | 152 | 27 | 37 |
| *Gasparinisaura cincolsaltensis* | Ornithopoda | NMST 20392 | 50 | 7 | 10 |
| *Iguanodon bernissartensis* | Ornithopoda | BINS 1534 | 663 | 111 | 140 |
| *Iguanodon bernissartensis* | Ornithopoda | BINS 1536 | 613 | 158 | 153 |
| *Iguanodon bernissartensis* | Ornithopoda | BINS 1562 | 668 | 149 | 158 |
| *Iguanodon bernissartensis* | Ornithopoda | BINS 1639 | 590 | 129 | 178 |
| *Iguanodon bernissartensis* | Ornithopoda | BINS 1657 | 500 | 70 | 185 |
| *Iguanodon bernissartensis* | Ornithopoda | BINS 1715 | 745 | 106 | 141 |
| *Mattaburrasaurus langdoni* | Ornithopoda | Fukui *Muttaburrasaurus* | 620 | 103 | 135 |
| *Ouranosaurus nigeriensis* | Ornithopoda | Fukui *Ouranosaurus* | 415 | 72 | 91 |
| *Tenontosaurus tilletti* | Ornithopoda | AMNH 3014 | 193 | 38 | 56 |
| *Tenontosaurus tilletti* | Ornithopoda | AMNH 3022 | 130 | 19 | 39 |
| *Tenontosaurus tilletti* | Ornithopoda | AMNH 3043 | 200 | 50 | 60 |
| *Tenontosaurus tilletti* | Ornithopoda | YPM BB1 | 215 | 48 | 70 |
| *Pachycephalosaurus wyomingensis* | Marginocephalia | Fukui *Pachycephalosaurus* | 157 | 27 | 42 |
| *Protoceratops andrewsi* | Marginocephalia | AMNH 6424 | 146 | 25 | 48 |
| Ceratopsia indet. | Marginocephalia | CMN 41628 | 165 | 27 | 58 |
| *Leptoceratops gracilis* | Marginocephalia | AMNH 5205 | 225 | 57 | 75 |
| *Leptoceratops gracilis* | Marginocephalia | CMN 8889 | 200 | 28 | 55 |

**Table S4. Specimens, phylogenetic groupings and measurements used in regressions of radius proportions.**

| **Taxon** | **Grouping** | **Specimen number** | **Length (mm)** | **AP width (mm)** | **ML width (mm)** |
| --- | --- | --- | --- | --- | --- |
| *Edmontonia longiceps* | Ankylosauria | AMNH 5381 | 565 | 83 | 52 |
| *Edmontonia longiceps* | Ankylosauria | CMN 8531 | 293 | 68 | 61 |
| *Euoplocephalus tutus* | Ankylosauria | AMNH 5403 | 308 | 58 | 78 |
| *Euoplocephalus tutus* | Ankylosauria | AMNH 5404 | 275 | 49 | 87 |
| *Sauropelta edwardsi* | Ankylosauria | AMNH 3016 | 355 | 55 | 67 |
| *Sauropelta edwardsi* | Ankylosauria | AMNH 3035 | 380 | 50 | 96 |
| *Sauropelta edwardsi* | Ankylosauria | AMNH 5833 | 290 | 43 | 57 |
| *Heterodontosaurus tucki* | Bipedal ornithischian | USNM 299765 | 57 | 4 | 8 |
| *Agilisaurus louderbacki* | Bipedal ornithischian | Fukui *Agilisaurus* | 68 | 8 | 16 |
| *Parksosaurus warreni* | Bipedal ornithischian | ROM 804 | 128 | 12 | 13 |
| *Thescelosaurus neglectus* | Bipedal ornithischian | AMNH 5034 | 195 | 24 | 22 |
| *Thescelosaurus neglectus* | Bipedal ornithischian | NMST *Thescelosaurus* | 143 | 16 | 18 |
| *Centrosaurus apertus* | Ceratopsidae | ROM 1426 | 305 | 48 | 44 |
| *Centrosaurus apertus* | Ceratopsidae | ROM 767 | 335 | 44 | 36 |
| Ceratopsidae indet. | Ceratopsidae | CMN 8547 | 130 | 55 | 77 |
| Ceratopsidae indet. | Ceratopsidae | TMP 82.18.96 | 225 | 31 | 30 |
| Ceratopsidae indet. | Ceratopsidae | TMP 92.36.783 | 290 | 44 | 56 |
| Ceratopsidae indet. | Ceratopsidae | TMP 97.12.220 | 210 | 39 | 78 |
| *Chasmosaurus belli* | Ceratopsidae | CMN 2245 | 360 | 39 | 29 |
| *Chasmosaurus* sp. | Ceratopsidae | ROM 839 | 340 | 45 | 63 |
| *Chasmosaurus belli* | Ceratopsidae | ROM 843 | 350 | 53 | 59 |
| *Pachyrhinosaurus canadensis* | Ceratopsidae | TMP 86.55.264 | 225 | 31 | 35 |
| *Pachyrhinosaurus canadensis* | Ceratopsidae | TMP 89.55.1313 | 205 | 28 | 18 |
| *Pachyrhinosaurus canadensis* | Ceratopsidae | TMP 89.55.1339 | 345 | 57 | 31 |
| *Pachyrhinosaurus canadensis* | Ceratopsidae | TMP 89.55.1496 | 300 | 52 | 31 |
| *Triceratops horridus* | Ceratopsidae | AMNH 5880 | 480 | 90 | 110 |
| *Triceratops horridus* | Ceratopsidae | CM 1618 | 405 | 72 | 44 |
| *Brachylophosaurus canadensis* | Hadrosauridae | CMN 8893 | 660 | 40 | 66 |
| *Corythosaurus casuarius* | Hadrosauridae | CMN 8704 | 620 | 44 | 52 |
| *Corythosaurus casuarius* | Hadrosauridae | USNM 11893 | 395 | 27 | 52 |
| *Corythosaurus casuarius* | Hadrosauridae | USNM 358560 | 530 | 31 | 30 |
| *Edmontosaurus regalis* | Hadrosauridae | CMN 2289 | 665 | 60 | 78 |
| *Edmontosaurus* sp. | Hadrosauridae | CMN 419 | 640 | 50 | 59 |
| *Edmontosaurus annectens* | Hadrosauridae | ROM 801 | 633 | 50 | 74 |
| *Edmontosaurus* sp. | Hadrosauridae | USNM 3814 | 565 | 52 | 83 |
| *Gryposaurus notabilis* | Hadrosauridae | ROM 764 | 550 | 46 | 66 |
| *Gryposaurus notabilis* | Hadrosauridae | USNM 214577 | 365 | 20 | 16 |
| Hadrosauridae indet. | Hadrosauridae | ROM 829 | 565 | 34 | 48 |
| Hadrosauridae indet. | Hadrosauridae | TMP 90.36.421 | 450 | 22 | 48 |
| Hadrosauridae indet. | Hadrosauridae | TMP 92.36.413 | 240 | 17 | 25 |
| *Hadrosaurus foulkii* | Hadrosauridae | AMNH 5472 | 435 | 32 | 36 |
| *Hadrosaurus foulkii* | Hadrosauridae | CMN 40603 | 500 | 36 | 64 |
| *Hadrosaurus foulkii* | Hadrosauridae | TMP 84.181.1 | 515 | 50 | 59 |
| *Hypacrosaurus altispinus* | Hadrosauridae | AMNH 5272 | 685 | 55 | 71 |
| *Hypacrosaurus altispinus* | Hadrosauridae | AMNH 5357 | 475 | 38 | 53 |
| *Hypacrosaurus altispinus* | Hadrosauridae | USNM 11950 | 410 | 26 | 57 |
| *Maiasaura peeblesorum* | Hadrosauridae | ROM 44770 | 568 | 42 | 50 |
| *Maiasaura peeblesorum* | Hadrosauridae | ROM 44771 | 185 | 17 | 14 |
| *Maiasaura peeblesorum* | Hadrosauridae | YPM PU22400 | 73 | 4 | 7 |
| *Nipponsaurus sachalinensis* | Hadrosauridae | NMST *Nipponosaurus* | 250 | 20 | 32 |
| *Parasaurolophus walkeri* | Hadrosauridae | ROM 768 | 495 | 48 | 77 |
| *Parasaurolophus walkeri* | Hadrosauridae | TMP 92.53.21 | 620 | 42 | 57 |
| *Prosaurolophus maximus* | Hadrosauridae | ROM 1423 | 575 | 46 | 81 |
| *Prosaurolophus maximus* | Hadrosauridae | ROM 787 | 470 | 46 | 40 |
| *Saurolophus osborni* | Hadrosauridae | AMNH 5271 | 438 | 41 | 33 |
| *Stegosaurus armatus* | Stegosauria | CM 36700 | 605 | 84 | 105 |
| *Stegosaurus armatus* | Stegosauria | USNM 4937 | 452 | 66 | 109 |
| *Stegosaurus armatus* | Stegosauria | USNM 5804 | 429 | 68 | 99 |
| *Stegosaurus armatus* | Stegosauria | USNM 6646 | 465 | 69 | 63 |
| *Stegosaurus armatus* | Stegosauria | USNM 7401 | 334 | 42 | 58 |
| *Stegosaurus armatus* | Stegosauria | YPM 1365/1855/6 | 415 | 70 | 104 |
| *Stegosaurus armatus* | Stegosauria | YPM 4834/5 | 395 | 61 | 109 |
| *Stegosaurus armatus* | Stegosauria | YPM 4835 | 385 | 63 | 114 |
| *Stegosaurus armatus* | Stegosauria | YPM 4836 | 460 | 64 | 108 |
| *Tuojiangosaurus multispinus* | Stegosauria | Fukui *Tuojiangosaurus* | 358 | 70 | 89 |
| *Iguanodon bernissartensis* | Ornithopoda | BINS 1534 | 518 | 67 | 80 |
| *Iguanodon bernissartensis* | Ornithopoda | BINS 1536 | 505 | 62 | 86 |
| *Iguanodon bernissartensis* | Ornithopoda | BINS 1562 | 515 | 69 | 112 |
| *Iguanodon bernissartensis* | Ornithopoda | BINS 1639 | 503 | 50 | 114 |
| *Iguanodon bernissartensis* | Ornithopoda | BINS 1657 | 505 | 82 | 81 |
| *Iguanodon bernissartensis* | Ornithopoda | BINS 1715 | 588 | 71 | 78 |
| *Mantellisaurus atherfieldensis* | Ornithopoda | BINS 1551 | 345 | 40 | 56 |
| *Bactrosaurus johnsoni* | Ornithopoda | Fukui *Bactrosaurus* | 215 | 19 | 28 |
| *Muttaburrasaurus langdoni* | Ornithopoda | Fukui *Muttaburrasaurus* | 490 | 52 | 67 |
| *Ouranosaurus nigeriensis* | Ornithopoda | Fukui *Ouranosaurus* | 340 | 37 | 66 |
| *Gasparinisaura cincolsaltensis* | Ornithopoda | NMST 20392 | 44 | 4 | 7 |
| *Camptosaurus dispar* | Ornithopoda | AMNH 586 | 270 | 33 | 38 |
| *Camptosaurus dispar* | Ornithopoda | AMNH 596 | 260 | 34 | 35 |
| *Camptosaurus dispar* | Ornithopoda | USNM 4282 | 230 | 28 | 35 |
| *Camptosaurus dispar* | Ornithopoda | USNM 4697 | 240 | 32 | 29 |
| *Camptosaurus dispar* | Ornithopoda | USNM 5473 | 255 | 28 | 32 |
| *Camptosaurus dispar* | Ornithopoda | YPM 6794 | 183 | 25 | 26 |
| *Tenontosaurus tilletti* | Ornithopoda | USNM 466042 | 210 | 22 | 38 |
| *Tenontosaurus tilletti* | Ornithopoda | AMNH 3014 | 185 | 18 | 21 |
| *Tenontosaurus tilletti* | Ornithopoda | YPM BB1 | 200 | 19 | 22 |
| *Protoceratops andrewsi* | Marginocephalia | AMNH 6424 | 135 | 11 | 16 |
| *Leptoceratops gracilis* | Marginocephalia | CMN 8889 | 165 | 20 | 23 |
| *Pachycephalosaurus wyomingensis* | Marginocephalia | Fukui *Pachycephalosaurus* | 147 | 16 | 21 |

**3. Stratigraphic Data**

**Table S5. Data used for branch length calculations for Independent Contrasts**

| **Taxon** | **Formation** | **Age** | **FAD** | **LAD** | **Data source** | **Group** |
| --- | --- | --- | --- | --- | --- | --- |
| *Ankylosaurus magniventris* | Hell Creek Formation (Montana); Lance Formation, Scollard Formation | late Maastrichtian | 70.6 | 65.5 | Vickaryous et al. 2004 | Ankylosauria |
| *Euoplocephalus tutus* | Upper Two Medicine Formation, Dinosaur Park Formation, Horseshoe Canyon Formation | late Campanian - early Maastrichtian | 83.5 | 65.5 | Vickaryous et al. 2004 | Ankylosauria |
| *Gastonia burgei* | Lower Cedar Mountain Formation | Berriasian-Hauterivian | 146 | 130 | Vickaryous et al. 2004 | Ankylosauria |
| *Edmontonia longiceps* | Judith River Formation; Lance Formation, Dinosaur Park Formation, Horseshoe Canyon Formation, St Mary River Formation | late Campanian - Maastrichtian | 83.5 | 65.5 | Vickaryous et al. 2004 | Ankylosauria |
| *Panoplosaurus mirus* | Dinosaur Park Formation | late Campanian | 83.5 | 70.6 | Vickaryous et al. 2004 | Ankylosauria |
| *Sauropelta edwardsi* | Cloverly Formation | late Aptian | 125 | 112 | Vickaryous et al. 2004 | Ankylosauria |
| *Struthiosaurus austriacus* | Gosau Formation | Campanian | 83.5 | 70.6 | Vickaryous et al. 2004 | Ankylosauria |
| *Centrosaurus apertus* | Dinosaur Park Formation | late Campanian | 83.5 | 70.6 | Dodson et al. 2004 | Ceratopsidae |
| *Pachyrhinosaurus canadensis* | Horseshoe Canyon Formation, St Mary River Formation, Prince Creek Formation, Dinosaur Park Formation | Campanian-Maastrichtian | 83.5 | 65.5 | Dodson et al. 2004; Ryan et al. 2010 | Ceratopsidae |
| *Styracosaurus albertensis* | Dinosaur Park Formation | late Campanian | 83.5 | 70.6 | Dodson et al. 2004 | Ceratopsidae |
| *Chasmosaurus belli* | Dinosaur Park Formation | late Campanian | 83.5 | 70.6 | Dodson et al. 2004 | Ceratopsidae |
| *Triceratops horridus* | Lance Formation, Evanston Formation, Hell Creek Formation (Montana & South Dakota & North Dakota), Laramie Formation, Scollard Formation, Frenchman Formation | late Maastrichtian | 70.6 | 65.5 | Dodson et al. 2004 | Ceratopsidae |
| *Hadrosaurus foulkii* | Merchantville Formation; Woodbury Formation; Marshalltown Formation | Campanian | 83.5 | 70.6 | Horner et al. 2004 | Hadrosauridae |
| *Parasaurolophus walkeri* | Dinosaur Park Formation | late Campanian | 83.5 | 70.6 | Horner et al. 2004 | Hadrosauridae |
| *Hypacrosaurus altispinus* | Horseshoe Canyon Formation | early Maastrichtian | 70.6 | 65.5 | Horner et al. 2004 | Hadrosauridae |
| *Lambeosaurus lambei* | Dinosaur Park Formation | late Campanian | 83.5 | 70.6 | Horner et al. 2004 | Hadrosauridae |
| *Corythosaurus casuarius* | Dinosaur Park Formation | late Campanian | 83.5 | 70.6 | Horner et al. 2004 | Hadrosauridae |
| *Brachylophosaurus canadensis* | Oldman Formation, Dinosaur Park Formation, Judith River Formation | late Campanian | 83.5 | 70.6 | Horner et al. 2004 | Hadrosauridae |
| *Maiasaura peeblesorum* | Upper Two Medicine Formation | middle-late Campanian | 83.5 | 70.6 | Horner et al. 2004 | Hadrosauridae |
| *Edmontosaurus annectens* | Scollard Formation, Hell Creek Formation, Lance Formation, Laramie Formation | late Maastrichtian | 70.6 | 65.5 | Horner et al. 2004 | Hadrosauridae |
| *Edmontosaurus regalis* | Horseshoe Canyon Formation; St Mary River Formation, Scollard Formation, Hell Creek Formation, Lance Formation, Laramie Formation | early - late Maastrichtian | 70.6 | 65.5 | Horner et al. 2004 | Hadrosauridae |
| *Prosaurolophus maximus* | Dinosaur Park Formation | late Campanian | 83.5 | 70.6 | Horner et al. 2004 | Hadrosauridae |
| *Saurolophus osborni* | Horseshoe Canyon Formation | early Maastrichtian | 70.6 | 65.5 | Horner et al. 2004 | Hadrosauridae |
| *Gryposaurus notabilis* | Dinosaur Park Formation | late Campanian | 83.5 | 70.6 | Horner et al. 2004; Prieto-Márquez 2010 | Hadrosauridae |
| *Stegoceras validum* | Oldman Formation, Dinosaur Park Formation, Horseshoe Canyon Formation (Alberta), Judith River Formation | late Campanian | 83.5 | 70.6 | Maryanska et al. 2004 | Marginocephalia |
| *Pachycephalosaurus wyomingensis* | Lance Formation, Hell Creek Formation (South Dakota & Montana), Judith River Formation | late Maastrichtian | 70.6 | 65.5 | Maryanska et al. 2004 | Marginocephalia |
| *Yinlong downsi* | Upper Shishugou Formation | Oxfordian | 161 | 156 | Xu et al. 2006 | Marginocephalia |
| *Psittacosaurus mongoliensis* | Khukhtek Formation, Khulsyngol Formation, Shestakov Formation, Jiufotang Formation | Aptian-Albian | 125 | 99.6 | Sereno 2010; You & Dodson 2004 | Marginocephalia |
| *Psittacosaurus neimongoliensis* | Ejinhoro Formation | Early Cretaceous | 146 | 99.6 | Sereno 2010; You & Dodson 2004 | Marginocephalia |
| *Psittacosaurus lujiatunensis* | Lower Yixian Formation | Barremian | 130 | 125 | Sereno 2010 | Marginocephalia |
| *Protoceratops andrewsi* | Djadokhta Formation (Mongolia & China), Minhe Formation | late Santonian - early Campanian | 85.8 | 70.6 | You & Dodson 2004 | Marginocephalia |
| *Leptoceratops gracilis* | Scollard Formation, Lance Formation (Wyoming), Hell Creek Formation (Montana) | late Maastrichtian | 70.6 | 65.5 | You & Dodson 2004 | Marginocephalia |
| *Montanoceratops cerorhynchus* | St Mary River Formation; Horseshoe Canyon Formation | early Maastrichtian | 70.6 | 65.5 | You & Dodson 2004 | Marginocephalia |
| *Lesothosaurus diagnosticus* | Upper Elliot Formation | Hettangian-Sinemurian | 200 | 190 | Norman et al. 2004a | Ornithischia |
| *Abrictosaurus consors* | Upper Elliot Formation | Hettangian-Sinemurian | 200 | 190 | Norman et al. 2004b | Ornithischia |
| *Heterodontosaurus tucki* | Upper Elliot Formation; Clarens Formation | Hettangian-Sinemurian | 200 | 190 | Norman et al. 2004b | Ornithischia |
| *Agilisaurus louderbacki* | Lower Shaximiao Formation | Bathonian-Callovian | 168 | 161 | Norman et al. 2004b | Ornithischia |
| *Othneilosaurus consors* | Morrison Formation | Kimmeridgian - Tithonian | 156 | 146 | Norman et al. 2004b | Ornithischia |
| *Gilmoreosaurus mongoliensis* | Iren Dabasu Formation | Campanian | 83.5 | 70.6 | Horner et al. 2004 | Ornithopoda |
| *Bactrosaurus johnsoni* | Iren Dabasu Formation | Campanian | 83.5 | 70.6 | Horner et al. 2004 | Ornithopoda |
| *Ouranosaurus nigeriensis* | Elrhaz Formation | late Aptian | 125 | 112 | Norman 2004 | Ornithopoda |
| *Mantellisaurus atherfieldensis* | Wessex Formation; Vectis Formation; Weald Clay, Lower Greensand; Wealden; etc. | Valanginian - Aptian | 140 | 112 | Norman 2004 | Ornithopoda |
| *Iguanodon bernissartensis* | Wessex Formation; Hastings beds; Weald Clay; Potton Sands; etc. | Valanginian - Albian | 140 | 99.6 | Norman 2004 | Ornithopoda |
| *Uteodon aphanoecetes* | Morrison Formation | Kimmeridgian - Tithonian | 156 | 146 | Carpenter & Wilson 2008 | Ornithopoda |
| *Camptosaurus dispar* | Morrison Formation | Kimmeridgian - Tithonian | 156 | 146 | Norman 2004 | Ornithopoda |
| *Dryosaurus altus* | Morrison Formation | Kimmeridgian - Tithonian | 156 | 146 | Norman 2004 | Ornithopoda |
| *Tenontosaurus tilleti* | Cloverly Formation; Paluxy Formation | late Aptian - middle Albian | 125 | 99.6 | Norman 2004 | Ornithopoda |
| *Muttaburrasaurus langdoni* | Mackunda Formation | Albian | 112 | 99.6 | Norman 2004 | Ornithopoda |
| *Gasparinisaura cincolsaltensis* | Rio Colorado Formation | Santonian-early Campanian | 85.8 | 70.6 | Norman et al. 2004b | Ornithopoda |
| *Hypsilophodon foxii* | Wessex Formation; Vectis Formation; Camarillas Formation, Encisco Group | Barremian | 130 | 125 | Norman et al. 2004b | Ornithopoda |
| *Parksosaurus warreni* | Horseshoe Canyon Formation, Scollard Formation | Maastrichtian | 70.6 | 65.5 | Norman et al. 2004b | Ornithopoda |
| *Thescelosaurus neglectus* | Lance Formation, Hell Creek Formation, Laramie Formation, Scollard Formation, Horseshoe Canyon Formation, Frenchman Formation | late Campanian-late Maastrichtian | 83.5 | 65.5 | Norman et al. 2004b | Ornithopoda |
| *Dacentrurus armatus* | Kimmeridge Clay | Kimmeridgian-Tithonian | 156 | 146 | Galton & Upchurch 2004; Maidment et al. 2008 | Stegosauria |
| *Stegosaurus armatus* | Morrison Formation | Kimmeridgian-Tithonian | 156 | 146 | Galton & Upchurch 2004; Maidment et al. 2008 | Stegosauria |
| *Loricatosaurus priscus* | Lower Oxford Clay | early-middle Callovian | 165 | 161 | Galton & Upchurch 2004 | Stegosauria |
| *Kentrosaurus aethiopicus* | Tendaguru Formation | Kimmeridgian | 156 | 151 | Galton & Upchurch 2004 | Stegosauria |
| *Huayangosaurus taibaii* | Lower Shaximiao Formation | Bathonian-Callovian | 168 | 161 | Galton & Upchurch 2004 | Stegosauria |
| *Tuojiangosaurus multispinus* | Upper Shaximiao Formation | Late Jurassic | 161 | 146 | Galton & Upchurch 2004 | Stegosauria |
| *Scelidosaurus harrisonii* | Lower Lias | late Sinemurian | 197 | 190 | Norman et al. 2004c | Thyreophora |

**References**

Carpenter K, Wilson Y (2008) A new species of *Camptosaurus* (Ornithopoda: Dinosauria) from the Morrison Formation (Upper Jurassic) of Dinosaur National Monument, Utah, and a biomechanical analysis of its forelimb. Annals of the Carnegie Museum of Natural History 76: 227–265.

Dodson P, Forster CA, Sampson SD (2004) Ceratopsidae. In: Weishampel DB, Dodson P, Osmólska H, editors. The Dinosauria (second edition). Berkeley: University of California Press. pp. 494–516.

Galton PM, Upchurch P (2004) Stegosauria. In: Weishampel DB, Dodson P, Osmólska H, editors. The Dinosauria (second edition). Berkeley: University of California Press. pp. 343–362.

Horner JR, Weishampel DB, Forster CA (2004) Hadrosauridae. In: Weishampel DB, Dodson P, Osmólska H, editors. The Dinosauria (second edition). Berkeley: University of California Press. pp. 438–463.

Maidment SCR, Norman DB, Barrett PM, Upchurch P (2008) Systematics and phylogeny of Stegosauria (Dinosauria: Ornithischia). J Syst Palaeontol 6: 364–407.

Maryańska T, Chapman RE, Weishampel DB (2004). Pachycephalosauria. In: Weishampel DB, Dodson P, Osmólska H, editors. The Dinosauria (second edition). Berkeley: University of California Press. pp. 464–477.

Norman DB (2004) Basal Iguanodontia. In: Weishampel DB, Dodson P, Osmólska H, editors. The Dinosauria (second edition). Berkeley: University of California Press. pp. 413–437.

Norman DB, Witmer LM, Weishampel DB (2004a) Basal Ornithischia. In: Weishampel DB, Dodson P, Osmólska H, editors. The Dinosauria (second edition). Berkeley: University of California Press. pp. 325–334.

Norman DB, Sues HD, Witmer LM, Coria, RA (2004b) Basal Ornithopoda. In: Weishampel DB, Dodson P, Osmólska H, editors. The Dinosauria (second edition). Berkeley: University of California Press. pp. 393–412.

Norman DB, Witmer LM, Weishampel DB (2004c) Basal Thyreophora. In: Weishampel DB, Dodson P, Osmólska H, editors. The Dinosauria (second edition). Berkeley: University of California Press. pp. 335–342.

Prieto-Márquez A (2010) Global phylogeny of Hadrosauridae (Dinosauria: Ornithopoda) using parsimony and baysian methods. Zool J Linn Soc-Lond 159: 435–503.

Ryan MJ, Eberth DA, Brinkman DB, Currie PJ, Tanke DH (2010) A new *Pachyrhinosaurus*-like ceratopsid from the Upper Dinosaur Park Formation (Late Campanian) of southern Alberta, Canada. In: Ryan MJ, Chinnery-Allegeier BJ, Eberth DA, editors. New Perspectives on Horned Dinosaurs. Bloomington: Indiana. pp. 141–155.

Sereno PC (2010) Taxonomy, cranial morphology, and relationships of parrot-beaked dinosaurs (Ceratopsia: Psittacosaurus). In: Ryan MJ, Chinnery-Allegeier BJ, Eberth DA, editors. New Perspectives on Horned Dinosaurs. Bloomington: Indiana. pp. 21–58.

Vickaryous MK, Maryańska T, Weishampel DB (2004) Ankylosauria. In: Weishampel DB, Dodson P, Osmólska H, editors. The Dinosauria (second edition). Berkeley: University of California Press. pp. 363–393.

Xu X, Forster CA, Clark JM, Mo J-Y (2006) A basal ceratopsian with transitional features from the Late Jurassic of northwestern China. P Roy Soc B-Biol Sci 273: 2135–2140.

You H, Dodson P (2004) Basal Ceratopsia. In: Weishampel DB, Dodson P, Osmólska H, editors. The Dinosauria (second edition). Berkeley: University of California Press. pp. 494–516.

**4. K-means cluster analysis**

**Table S6. K-means cluster analysis.** Five iterations were carried out. Numbers 1–4 correspond to different clusters.

| **Taxon** | **Grouping** | **Specimen number** | **Iteration 1** | **Iteration 2** | **Iteration 3** | **Iteration 4** | **Iteration 5** |
| --- | --- | --- | --- | --- | --- | --- | --- |
| *Edmontonia longiceps* | Ankylosauria | CMN 8531 | 2 | 1 | 4 | 4 | 1 |
| *Euoplocephalus tutus* | Ankylosauria | AMNH 5403 | 2 | 1 | 4 | 4 | 1 |
| *Euoplocephalus tutus* | Ankylosauria | AMNH 5404 | 1 | 2 | 3 | 3 | 3 |
| *Sauropelta edwardsi* | Ankylosauria | AMNH 3016 | 1 | 2 | 3 | 3 | 3 |
| *Sauropelta edwardsi* | Ankylosauria | AMNH 3032 | 1 | 2 | 3 | 3 | 3 |
| *Sauropelta edwardsi* | Ankylosauria | AMNH 3035 | 2 | 1 | 4 | 4 | 1 |
| *Dacentrurus armatus* | Stegosauria | NHMUK 46013 | 2 | 1 | 4 | 4 | 1 |
| Stegosauria indet. | Stegosauria | NHMUK R5902 | 1 | 2 | 3 | 3 | 3 |
| *Stegosaurus armatus* | Stegosauria | USNM 4937 | 1 | 2 | 3 | 3 | 3 |
| *Stegosaurus armatus* | Stegosauria | SMA DS-RCR-2003-02 | 2 | 1 | 4 | 4 | 1 |
| *Stegosaurus mjosi* | Stegosauria | SMA 0092 | 2 | 1 | 3 | 3 | 3 |
| *Stegosaurus mjosi* | Stegosauria | SMA VF01 | 1 | 2 | 3 | 3 | 3 |
| *Stegosaurus mjosi* | Stegosauria | SMA 0017 | 2 | 1 | 3 | 3 | 3 |
| *Tuojiangosaurus multispinus* | Stegosauria | Fukui *Tuojiangosaurus* | 2 | 1 | 4 | 4 | 1 |
| *Leptoceratops gracilis* | Marginocephalia | CMN 8889 | 2 | 1 | 4 | 4 | 1 |
| *Pachycephalosaurus wyomingensis* | Marginocephalia | Fukui *Pachycephalosaurus* | 1 | 2 | 3 | 3 | 3 |
| *Protoceratops andrewsi* | Marginocephalia | AMNH 6424 | 2 | 1 | 4 | 4 | 1 |
| *Protoceratops andrewsi* | Marginocephalia | IVPP unregistered | 1 | 2 | 3 | 3 | 3 |
| *Psittacosaurus lujiatunensis* | Marginocephalia | IVPP V14341.1 | 2 | 1 | 4 | 4 | 1 |
| *Psittacosaurus lujiatunensis* | Marginocephalia | IVPP V14341.3 | 2 | 1 | 4 | 4 | 1 |
| *Psittacosaurus lujiatunensis* | Marginocephalia | IVPP V14341.4 | 2 | 1 | 4 | 4 | 1 |
| *Psittacosaurus lujiatunensis* | Marginocephalia | IVPP V14341.5 | 1 | 2 | 3 | 3 | 3 |
| *Psittacosaurus neimongoliensis* | Marginocephalia | IVPP 120888 | 2 | 1 | 4 | 4 | 1 |
| *Stegoceras validum* | Marginocephalia | UALVP 2 | 2 | 1 | 4 | 4 | 1 |
| *Yinlong downsi* | Marginocephalia | IVPP 14530 | 3 | 4 | 4 | 4 | 1 |
| *Centrosaurus apertus* | Ceratopsidae | ROM 767 | 2 | 1 | 4 | 4 | 1 |
| *Centrosaurus apertus* | Ceratopsidae | ROM 1426 | 2 | 1 | 4 | 4 | 1 |
| *Chasmosaurus belli* | Ceratopsidae | CMN 2245 | 1 | 2 | 3 | 3 | 3 |
| *Chasmosaurus* sp. | Ceratopsidae | ROM 839 | 2 | 1 | 4 | 4 | 1 |
| *Chasmosaurus belli* | Ceratopsidae | ROM 843 | 2 | 1 | 4 | 4 | 1 |
| *Vagaceratops irvinensis* | Ceratopsidae | CMN 41357 | 3 | 4 | 4 | 4 | 1 |
| *Pachyrhinosaurus* sp. | Ceratopsidae | TMP 2002.76.01 | 2 | 1 | 4 | 4 | 1 |
| *Styracosaurus albertensis* | Ceratopsidae | CMN 344 | 2 | 1 | 4 | 4 | 1 |
| *Triceratops horridus* | Ceratopsidae | CM 1618 | 2 | 1 | 4 | 4 | 1 |
| *Brachylophosaurus canadensis* | Hadrosauridae | CMN 8893 | 4 | 3 | 2 | 1 | 4 |
| *Brachylophosaurus canadensis* | Hadrosauridae | TMP 1990.104.01 | 4 | 3 | 2 | 1 | 4 |
| *Corythosaurus casuarius* | Hadrosauridae | ROM 1947 | 4 | 3 | 2 | 1 | 4 |
| *Corythosaurus casuarius* | Hadrosauridae | ROM 845 | 4 | 3 | 2 | 1 | 4 |
| *Corythosaurus casuarius* | Hadrosauridae | TMP 1980.40.01 | 4 | 3 | 2 | 1 | 4 |
| *Edmontosaurus regalis* | Hadrosauridae | CMN 2289 | 4 | 3 | 1 | 2 | 2 |
| *Edmontosaurus annectens* | Hadrosauridae | ROM 801 | 4 | 3 | 1 | 2 | 2 |
| *Gryposaurus notabilis* | Hadrosauridae | ROM 764 | 4 | 3 | 1 | 2 | 2 |
| *Gryposaurus notabilis* | Hadrosauridae | TMP 1980.22.01 | 1 | 2 | 1 | 2 | 2 |
| Hadrosauridae indet. | Hadrosauridae | CMN 40603 | 4 | 3 | 1 | 2 | 2 |
| *Hadrosaurus foulkii* | Hadrosauridae | TMP 84.181.1 | 4 | 3 | 1 | 2 | 2 |
| *Hypacrosaurus altispinus* | Hadrosauridae | AMNH 5357 | 4 | 3 | 2 | 1 | 4 |
| *Hypacrosaurus altispinus* | Hadrosauridae | USNM 11950 | 1 | 2 | 3 | 3 | 3 |
| *Hypacrosaurus altispinus* | Hadrosauridae | CMN 8501 | 4 | 3 | 2 | 1 | 4 |
| *Lambeosaurus lambei* | Hadrosauridae | ROM 1218 | 4 | 3 | 2 | 1 | 4 |
| *Maiasaura peeblesorum* | Hadrosauridae | ROM 44771 | 1 | 2 | 3 | 3 | 3 |
| *Nipponsaurus sachalinensis* | Hadrosauridae | NMST *Nipponosaurus* | 4 | 3 | 1 | 2 | 2 |
| *Parasaurolophus walkeri* | Hadrosauridae | ROM 768 | 4 | 3 | 1 | 2 | 2 |
| *Parasaurolophus walkeri* | Hadrosauridae | TMP 92.53.1 | 4 | 3 | 2 | 1 | 4 |
| *Prosaurolophus maximus* | Hadrosauridae | ROM 787 | 4 | 3 | 1 | 2 | 2 |
| *Saurolophus osborni* | Hadrosauridae | AMNH 5271 | 4 | 3 | 1 | 2 | 2 |
| *Bactrosaurus johnsoni* | Ornithopoda | Fukui *Bactrosaurus* | 4 | 3 | 1 | 2 | 2 |
| *Camptosaurus dispar* | Ornithopoda | AMNH 596 | 2 | 1 | 4 | 4 | 1 |
| *Camptosaurus dispar* | Ornithopoda | USNM 4282 | 2 | 1 | 4 | 4 | 1 |
| *Camptosaurus dispar* | Ornithopoda | USNM 5473 | 1 | 2 | 3 | 3 | 3 |
| *Camptosaurus dispar* | Ornithopoda | YPM 6794 | 1 | 2 | 3 | 3 | 3 |
| *Uteodon aphanoecetes* | Ornithopoda | CM 11337 | 2 | 1 | 4 | 4 | 1 |
| *Mantellisaurus atherfieldensis* | Ornithopoda | BINS 1551 | 1 | 2 | 3 | 3 | 3 |
| *Gasparinisaura cincolsaltensis* | Ornithopoda | NMST 20392 | 1 | 2 | 1 | 2 | 2 |
| *Iguanodon bernissartensis* | Ornithopoda | BINS 1534 | 2 | 1 | 4 | 4 | 1 |
| *Iguanodon bernissartensis* | Ornithopoda | BINS 1536 | 1 | 2 | 3 | 3 | 3 |
| *Iguanodon bernissartensis* | Ornithopoda | BINS 1562 | 2 | 1 | 4 | 4 | 1 |
| *Iguanodon bernissartensis* | Ornithopoda | BINS 1657 | 2 | 1 | 4 | 4 | 1 |
| *Iguanodon bernissartensis* | Ornithopoda | BINS 1715 | 2 | 1 | 4 | 4 | 1 |
| *Muttaburrasaurus langdoni* | Ornithopoda | Fukui *Muttaburrasaurus* | 1 | 2 | 3 | 3 | 3 |
| *Ouranosaurus nigeriensis* | Ornithopoda | Fukui *Ouranosaurus* | 1 | 2 | 3 | 3 | 3 |
| *Tenontosaurus tilletti* | Ornithopoda | AMNH 3014 | 1 | 2 | 3 | 3 | 3 |
| *Tenontosaurus tilletti* | Ornithopoda | USNM 466042 | 1 | 2 | 3 | 3 | 3 |
| *Tenontosaurus tilletti* | Ornithopoda | YPM BB1 | 1 | 2 | 3 | 3 | 3 |
| *Tenontosaurus tilletti* | Ornithopoda | MANCH LL 12275 | 1 | 2 | 3 | 3 | 3 |
| *Agilisaurus louderbacki* | Basal ornithischians | Fukui *Agilisaurus* | 2 | 1 | 4 | 4 | 1 |
| *Hypsilophodon foxii* | Basal ornithischians | NHMUK R196 | 1 | 2 | 3 | 3 | 3 |
| *Parksosaurus warreni* | Basal ornithischians | ROM 804 | 2 | 1 | 4 | 4 | 1 |
| *Thescelosaurus neglectus* | Basal ornithischians | AMNH 5034 | 2 | 1 | 4 | 4 | 1 |
| *Thescelosaurus neglectus* | Basal ornithischians | NMST *Thescelosaurus* | 2 | 1 | 4 | 4 | 1 |
| *Lesothosaurus diagnosticus* | Basal ornithischians | NHMUK RUB 17 | 2 | 1 | 4 | 4 | 1 |
| *Abrictosaurus consors* | Basal ornithischians | NHMUK RUB 54 | 2 | 1 | 3 | 3 | 3 |
